# Delta Inulin Adjuvant Enhances Plasmablast Generation, Expression of Activation-Induced Cytidine Deaminase and B-Cell Affinity Maturation in Human Subjects Receiving Seasonal Influenza Vaccine

**DOI:** 10.1371/journal.pone.0132003

**Published:** 2015-07-15

**Authors:** Lei Li, Yoshikazu Honda-Okubo, Connie Li, Dimitar Sajkov, Nikolai Petrovsky

**Affiliations:** 1 Vaxine Pty Ltd, Flinders Medical Centre, Bedford Park, Adelaide, Australia; 2 Australian Respiratory and Sleep Medicine Institute, Flinders Medical Centre, Bedford Park, Adelaide, Australia; 3 Department of Endocrinology, Flinders Medical Centre/Flinders University, Adelaide, Australia; The George Washington University School of Medicine and Health Sciences, UNITED STATES

## Abstract

There is a major need for new adjuvants to improve the efficacy of seasonal and pandemic influenza vaccines. Advax is a novel polysaccharide adjuvant based on delta inulin that has been shown to enhance the immunogenicity of influenza vaccine in animal models and human clinical trials. To better understand the mechanism for this enhancement, we sought to assess its effect on the plasmablast response in human subjects. This pilot study utilised cryopreserved 7 day post-vaccination (7dpv) peripheral blood mononuclear cell samples obtained from a subset of 25 adult subjects from the FLU006-12 trial who had been immunized intramuscularly with a standard dose of 2012 trivalent inactivated influenza vaccine (TIV) alone (n=9 subjects) or combined with 5mg (n=8) or 10mg (n=8) of Advax adjuvant. Subjects receiving Advax adjuvant had increased 7dpv plasmablasts, which in turn exhibited a 2-3 fold higher rate of non-silent mutations in the B-cell receptor CDR3 region associated with higher expression of activation-induced cytidine deaminase (AID), the major enzyme controlling BCR affinity maturation. Together, these data suggest that Advax adjuvant enhances influenza immunity in immunized subjects via multiple mechanisms including increased plasmablast generation, AID expression and CDR3 mutagenesis resulting in enhanced BCR affinity maturation and increased production of high avidity antibody. How Advax adjuvant achieves these beneficial effects on plasmablasts remains the subject of ongoing investigation.

**Trial Registration:**

Australia New Zealand Clinical Trials Register ACTRN12612000709842 https://www.anzctr.org.au/Trial/Registration/TrialReview.aspx?id=362709

## Introduction

Poor vaccine immunogenicity remains a major challenge in influenza vaccine development. Adjuvants are able to enhance vaccine immunogenicity and thereby increase influenza protection in low responder populations (reviewed in [1]). Nevertheless, the widespread adoption of adjuvants in influenza vaccines has been slow due to safety concerns of oil emulsion adjuvants [2,3] and poor understanding of how such adjuvants work [4]. Advax is a novel polysaccharide adjuvant based on semi-crystalline microparticles of delta inulin [5]. Advax has previously been shown to enhance seasonal and pandemic influenza vaccine protection in murine [6] or ferret models [7], respectively. Notably, when combined with a poorly immunogenic avian influenza antigen, Advax adjuvant reduced virus shedding and provided robust protection of immunized ferrets against H5N1-associated mortality and clinical disease [7]. Advax adjuvant also enhanced immunogenicity of influenza vaccine administered to pregnant dams, resulting in enhanced protection of their pups via increased breast milk transfer of protective antibodies [8]. Importantly Advax adjuvant has been shown to have similar beneficial effects on antibody production in humans, as shown in clinical trials of a pandemic influenza vaccine [9] and a hepatitis B vaccine [10]. With Advax adjuvant advancing towards late stage human trials, it is important to better understand the actions of this novel adjuvant and, in particular, the mechanism whereby it enhances humoral immunity. In this study we sought to characterize the effect of Advax on human plasmablasts [11] using cryopreserved 7dpv PBMC from a subset of subjects in a previously conducted seasonal influenza vaccine study (FLU006). The results reveal unique adjuvant-related effects on plasmablast frequency, AID gene expression and B-cell receptor usage when subjects that received TIV vaccine alone were compared to those that received vaccine formulated with Advax adjuvant.

## Methods

### Trial Design and Study Subjects

FLU006 was undertaken in 2012 as a randomized, blinded, parallel-group single-center study in Adelaide, Australia, to assess the administration of seasonal influenza vaccine, using different delivery routes and different vaccine formulations with some study results having been previously reported [12]. As detailed in the FLU006 study protocol (S1 Protocol), consenting study subjects were provided the opportunity to participate in a sub-study (FLU006-12) where additional blood samples were obtained weekly post-immunization to allow collection of peripheral blood mononuclear cells (PBMC) for cryopreservation for future studies into adjuvant effects on adaptive immunity. The FLU006-12 plasmablast substudy was performed using 7dpv PBMC samples available from 25 adult subjects ranging in age from 19 to 82 years who had received intramuscular injections via needle and syringe of TIV alone (n = 9), TIV+Advax 5mg (n = 8) or TIV+Advax 10mg (n = 8). The FLU006 study was approved by the Flinders Clinical Research Ethics Committee and is registered on the publicly accessible Australia New Zealand Clinical Trial Registry accessible at https://www.anzctr.org.au/ as ACTRN12612000709842. All participants provided their written consent to participate in the study.

### Vaccine Composition

FLU006 subjects had been immunized with southern hemisphere 2012 trivalent inactivated influenza vaccine (Fluvax, CSL Ltd, Melbourne Australia) which included inactivated A/California/07/2009 (H1N1), A/Perth/16/2009 (H3N2), and B/Brisbane/60/2008-like viruses alone or with DI adjuvant (Advax, Vaxine Pty Ltd, Adelaide, Australia). A single batch of Advax adjuvant was used in the clinical trial that had been manufactured and released under current Good Manufacturing Practice (cGMP) by Sypharma Pty Ltd, Melbourne Australia[5].

### PBMC Isolation and Cryopreservation

Human peripheral blood mononuclear cells (PBMCs) were isolated from fresh heparinized whole blood by standard density gradient centrifugation using Ficoll-Paque Plus (GE Healthcare) in Leucosep tubes (Greiner Bio-One) according to the product manual. After isolation, the PBMC were washed twice in RPMI 1640 medium, followed by centrifugation with 300×g at room temperature to pellet the cells. The pellet was broken by scraping the 15mL centrifuge tube on a tube rack several times, followed by addition of complete RPMI 1640 medium with 10% heat-inactivated neonatal bovine serum (NBS). Then an equal volume of cold cryopreservation buffer (NBS+ 20%DMSO) was added to the cell suspension drop-wise and mixed well by inverting several times. The cell suspension was then transferred to cryovials and frozen down by using freezing containers (Mr. Frosty) at -80°C, followed by liquid nitrogen storage 24h later.

### Hemagglutination Inhibition Assay

Hemagglutination inhibition (HI) titers were measured by HI assay with 1% guinea pig red blood cells, as previously described [9], using A/California/07/2009, A/Perth/16/2009, and B/Brisbane/60/2008-like virus strains. The titer was expressed as the reciprocal of the highest serum dilution inhibiting hemagglutination. Seroprotection was defined as a HI titer of 40 or greater and seroconversion as a 4-fold or greater increase in HI titer together with a final HI titer of 40 or greater.

### FACS and Cell Sorting

After rapid thawing and washing, PBMCs were suspended in FACS buffer (PBS with 1% BSA). Specific mAbs or corresponding isotype fluorescence minus one (FMO) were added and kept at 4°C in the dark for 30 minutes, followed by washing twice with FACS buffer. Antibodies for FACS staining include anti-CD19 PE-Cy7, anti-CD20 PerCP-Cy5.5, and anti-CD38 FITC (BD Pharmingen) and anti-CD27 APC (eBioscience) [13]. Flow cytometry was performed on a FACSCanto II (Becton Dickenson) and data were analyzed using FlowJo software (TreeStar, Inc.). For plasmablast sorting, stained samples were sorted using a BD FACSAria cell sorter equipped with a 70μm nozzle into tubes containing 1ml of Tri Regent (Sigma) for RNA extraction.

### RNA Extraction and RT-qPCR

RNA was extracted from cell lysate by TRI Reagent. After phase separation, the aqueous phase was transferred to new tubes followed by adding half volume of pure ethanol for precipitating RNA. Further purification was performed with RNeasy mini kit (Qiagen) per the product handbook. RNA concentration was measured using Nanodrop 2000 (Thermo Fisher Scientific). Quality controls included a nanodrop OD260/280 ratio of more than 1.8 and integrity of RNA was checked by agarose gel electrophoresis. Reverse transcription was performed with iScript cDNA Synthesis (Bio-Rad) and qPCR was carried out on ABI 7500 Fast thermal cycler (Life technologies) using iTaq SYBR Green Supermix with ROX (Bio-Rad). 2^-∆∆Cq^ method was used for relative expression level analysis. Primer sequences for qPCR were: AICDA Forward 5'-AAGAGGCGTGACAGTGCTAC-3' and Reverse 5'-TCCGAGATGTAGCGGAGGAA-3'; HPRT1 Forward 5'-TGACACTGGCAAAACAATGCA-3' and Reverse 5'-GGTCCTTTTCACCAGCAAGCT-3'; 18S Forward 5'-CGGCTACCACATCCAAGGAA-3' and Reverse 5'-GCTGGAATTACCGCGGCT-3'.

### Mutation Rate Analysis of Igh Genes

RNA from sorted plasmablasts was used for reverse transcription and PCR by using the OneStep RT-PCR Kit (Qiagen). RT primer for constant region of IgG and the forward primer mix for leader region of *Igh* gene were both used at the final concentration of 0.6μM. Amplification was done for 20 cycles. Then 20 cycles of nested PCR was done using the RT-PCR product and internal primers for the variable regions of IgG. After the nested PCR, the DNA was purified by QIAquick PCR Purification Kit (Qiagen) and then inserted into TOPO TA cloning vector pCR4-TOPO (Life Technologies). Primers used in this study for amplifying Ig VH genes were as previously described [13,14]. Primer sequences for human IgG one-step RT-PCR were: Forward 5'-ACAGGTGCCCACTCCCAGGTGCAG-3', 5'-AAGGTGTCCAGTGTGARGTGCAG-3', 5'-CCCAGATGGGTCCTGTCCCAGGTGCAG-3', 5'-CAAGGAGTCTGTTCCGAGGTGCAG-3', and Reverse 5'-GGAAGGTGTGCACGCCGCTGGTC-3'. Primer sequences for human IgG nested PCR were: Forward 5'-SARGTGCAGCTCGTGGAG-3', 5'-GAGGTGCAGCTGTTGGAG-3' and Reverse 5'-AGTAGTCCTTGACCAGGCAGCCCAG-3'. After transformation into TOP10 competent cells (Life Technologies), bacterial colonies were grown on Kanamycin^+^ agar plates. Then at least 20 colonies were randomly picked from each plate for mini-prep of plasmid DNA and sequencing. DNA sequences were analyzed online by using IMGT/HighV-Quest software for gene usage and mutation rate analysis.

### ELISPOT Assay

ELISPOT assays were performed according to previously described methods [13,15]. To quantitate influenza-specific antibody secreting cells (ASC), the multiscreen 96-well HTS plates (Millipore) were coated with TIV at 5μg/ml HA protein of each strain. Thawed PBMCs were re-suspended in complete RPMI 1640 culture medium, counted and plated at the density of 0.2×10^6^ cells per well and cultured overnight. The plates were then washed and probed with anti-human IgG-HRP, IgM-HRP or IgA-HRP. After a final wash the spots were developed with AEC Substrate set (BD Biosciences) and enumerated on an ELISPOT reader (Cellular Technology Ltd). The baseline background was subtracted to obtain the 7dpv ASC frequency.

### Analysis of Antibody Avidity

Interactions of inactivated A/California/07/2009 antigen and patient plasma were studied by surface plasmon resonance with a BIAcore X100 instrument at 25°C using running buffer HBS-EP+ (10mM HEPES, 0.15M NaCl, 3mM EDTA, 0.005% v/v surfactant P20, pH 7.4). Anti-human IgG was immobilized to a CM5 chip (GE Healthcare Bio-Sciences AB) in flow cell 2 (fc2) and the reference flow cell 1 (fc1) using wizard amine coupling method. A CM5 chip was activated with EDC/NHS for 7 minutes with the excess activated carboxyl groups were inactivated with ethanolamine for 7 minutes following immobilization of anti-human IgG was diluted in 10mM sodium acetate buffer pH 5.0. Plasma pooled from immunization groups was diluted (1/1000) in HBS-EP+ buffer and injected over fc2 at a flow rate of 5 μl/min. fc1 with no captured anti-human IgG on the reference surface. The level of captured human IgG for each capture experiment was 1500RU (±150). Sixty μl of inactivated A/California/07/2009 antigen (CSL, Australia) was injected at 10 μl/min over both the IgG-captured surface (fc2) and reference surface (fc1) followed by 10 mins dissociation time. The surface was regenerated with 3M MgCl_2_ at 10 μl/ml for 70 sec. Sensorgrams were corrected for binding to reference fc1 and for buffer effects (double reference subtracted). Binding changes were recorded in RU and were taken from the end of the association phases from each sensorgram. Dissociation data points from the dissociation phase were fitted to the one phase exponential decay equation for calculation of dissociation rate constants (kd, unit s^-1^) for each vaccination group. Comparison of the binding avidities of antibodies in different vaccination group was performed by measurement of the reciprocal (kd^-1^) of the dissociation rate constants.

### Statistical Analysis

As this was a pilot study, and the size of potential differences was not known, a formal power calculation was not considered warranted prior to commencing the study. However, in a previous human study that included Advax adjuvant [9], a sample size of 6–8 subjects per group was sufficient to see differences in antibody and T-cell responses between the adjuvanted and unadjuvanted vaccine groups, hence a similar number of subjects was used as the target sample size for the current study. The Area Under Curve analysis for the 7dpv plasmablast response comparison was performed using GraphPad Prism v5 using the trapezoid rule. Data sets in the study were analyzed for statistical significance with unpaired *t* test for comparison of group means after passing the normality test. *Igh* gene mutation rate comparison was performed using the Mann-Whitney U Test. Pearson method was used for correlation analysis. Comparison of differences between groups in Table 1 was performed using ANOVA, median test or chi-square test, as appropriate, using Prism. Significance is shown as * for *p* < 0.05 and ** for *p* < 0.01.

**Table 1:** Subject Characteristics—FLU006-12 Substudy.

	All subjects	TIV alone	+Advax 5mg	+Advax 10mg	
	n = 25	n = 9	n = 8	n = 8	P-value ^1^
**Age**, median (range)	64.5 (19–81)	65.0 (19–78)	63.5 (42–80)	65.5 (41–81)	0.51
**Gender**					
Male, n (%)	11 (42.3)	6 (60)	4 (50)	5 (62.5)	0.86
Female, n (%)	15 (57.7)	4 (40)	4 (50)	3 (37.5)	
**Race,** n (%)					n/a
Caucasian	25 (100)	10 (100)	8 (100)	8 (100)	
Other	0 (0)	0 (0)	0 (0)	0 (0)	
**Flu vaccine history**, n (%)					
In 2011	18 (69.2)	7 (70)	6 (75)	5 (62.5)	0.86
Within last three years	21 (80.8)	7 (70)	7 (87.5)	7 (87.5)	0.54

^1^ Differences were compared between groups using ANOVA, median test or chi-square test as appropriate.

## Results

### Advax Adjuvant Effects on the Antibody Response

Subjects who agreed to participate and provide additional blood samples for the sub-study were predominantly from an older Caucasian population (median age 64.5 years) and most had a history of a seasonal influenza vaccine within the previous 3 years (80.8%) (Table 1). There were no significant differences in baseline characteristics between substudy groups. The substudy itself had fewer subjects than the parent study and hence was not powered to detect differences in seroprotection and seroconversion rates between groups, which was the function of the larger parent study, which will be reported elsewhere. Given the limited sample size, the confidence intervals in seroprotection and seroconversion rates overlapped between groups in the sub-study, however point estimates showed a clear trend towards higher antibody titer increases in the Advax-adjuvanted groups, with the Advax 10mg dose group achieving the highest point estimates across all three vaccine strains for seroconversion, seroprotection and GMT fold increases across the vaccine strains (Table 2), and it is noted that H1 A/Cal and B/Bris showed more advantage. The trends seen in the substudy immunogenicity results are consistent with the findings in the larger parent FLU006 study, some results of which have already been reported [12].

**Table 2:** Seroprotection, seroconversion and GMT fold increase 28 days post immunization for subjects in the FLU006-12 substudy.

Groups	n		Day 0 GMT (95% CI)	Day 28 GMT (95% CI)	Day 28 Seroprotection %, (95% CI)	Day 28 Seroconversion %, (95% CI)	Day 28 GMT fold change (95% CI)
**TIV**		A/Cal	23.33 (8.68, 62.71)	80.00 (22.93, 279.1)	77.78 (44.28, 94.66)	33.33 (11.73, 64.91)	3.43 (1.19, 9.88)
	9	A/Per	21.6 (11.6, 40.22)	43.20 (26.35, 70.83)	88.89 (54.33, 99.99)	22.22 (5.34, 55.72)	2.00 (0.94, 4.25)
		B/Bris	10.00 (6.86, 14.58)	15.87 (10.01, 25.18)	11.11 (0.01, 45.67)	0.00 (0.00, 34.46)	1.59 (1.09, 2.31)
**Advax 5mg**		A/Cal	20.00 (9.36, 42.72)	61.69 (27.28, 139.5)	75.00 (40.09, 93.69)	37.5 (13.49, 69.62)	3.08 (1.01, 9.40)
	8	A/Per	23.78 (8.22, 68.78)	56.57 (21.24, 150.7)	62.50 (30.38, 86.51)	25.0 (6.31, 59.91)	2.38 (0.82, 6.88)
		B/Bris	10.00 (4.68, 21.36)	16.82 (9.23, 30.64)	12.50 (0.11, 49.22)	0.00 (0.00, 37.22)	1.68 (1.11, 2.53)
**Advax 10mg**		A/Cal	16.41 (8.92, 30.19)	88.33 (37.29, 209.2)	85.71 (46.65, 99.47)	71.43 (35.24, 92.44)	5.38 (1.59, 18.23)
	8	A/Per	16.41 (5.50, 48.93)	36.23 (13.21, 99.35)	71.43 (35.24, 92.44)	14.29 (0.53, 53.35)	2.21 (1.11, 4.38)
		B/Bris	13.46 (5.10, 35.48)	32.81 (14.69, 73.29)	57.14 (24.98, 84.25)	28.57 (7.56, 64.76)	2.44 (1.09, 5.45)

### Advax Adjuvant Enhances the 7dpv Plasmablast Response

**Fig 1:**
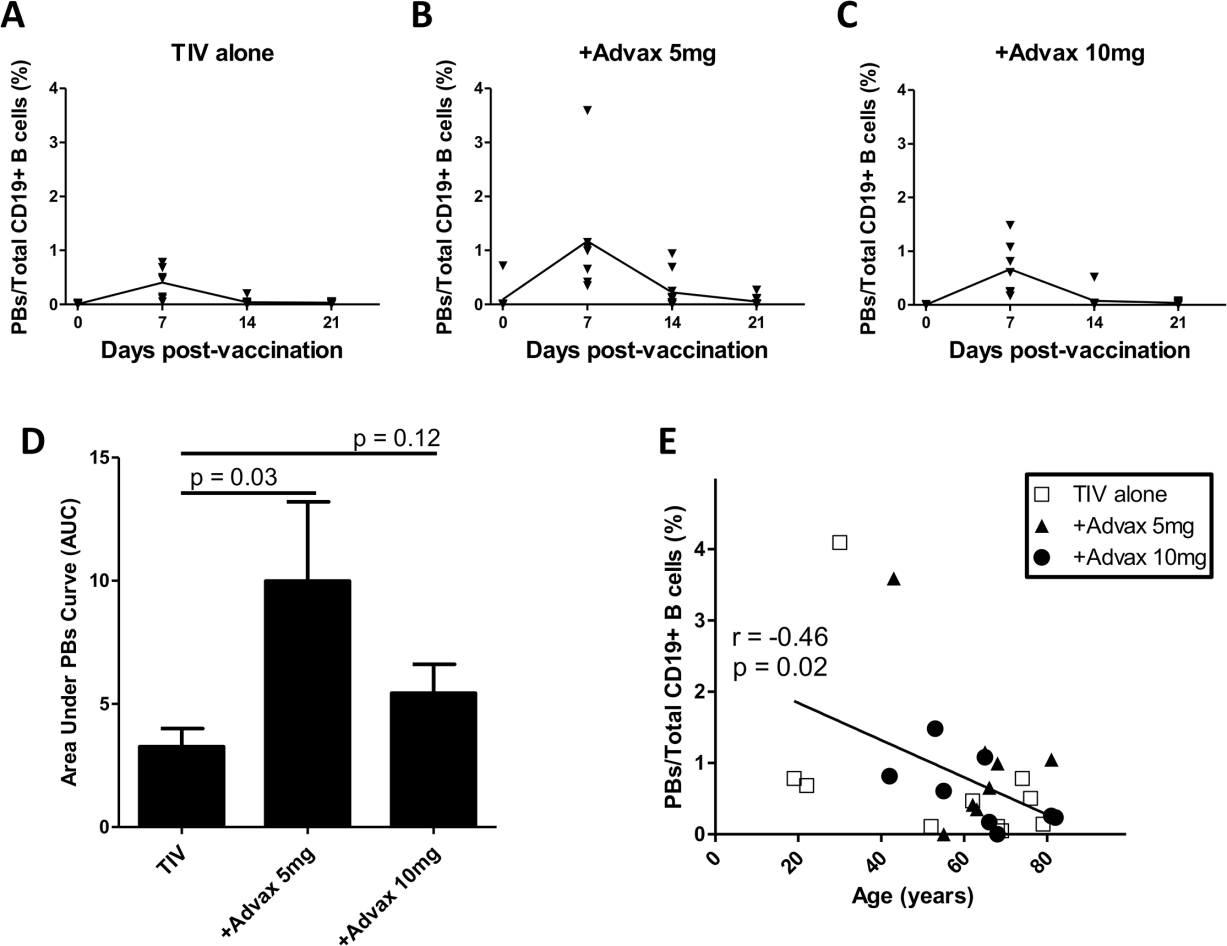
Advax adjuvant enhances plasmablast generation. Plasmablast frequency as a proportion of total CD19^+^ B cells measured by FACS (mean ± SEM), pre-immunization and 7, 14 and 21 days post-immunization in groups receiving TIV alone (A), TIV+Advax 5mg (B) or TIV+Advax 10mg (C). The area under plasmablast curve (AUC) was used as an estimate of total plasmablast generation post-immunization (mean AUC ± SEM) (D). Also shown is correlation between subject age and 7dpv plasmablast frequency (E).

Previous studies have reported a correlation between the peak frequency of 7dpv-plasmablasts and the 28dpv HI response to influenza immunization [16]. We therefore asked whether the higher antibody response in subjects that received Advax adjuvant was due to increased plasmablast generation. Plasmablasts in all groups were largely undetectable in peripheral blood pre-immunization, rose to a peak at 7dpv and then rapidly declined back to baseline by 21dpv (Fig 1A–1C). When the area under the post-immunization plasmablast curve was calculated (Fig 1D), the group that received Advax 5mg group had significantly increased total plasmablast generation (mean area 9.99, standard deviation (SD) 8.5, p = 0.03) when compared to the TIV alone group (mean area 3.27, SD 2.21). Plasmablast generation in the Advax 10mg group (mean area 5.44, SD 3.09) also had a higher point estimate but this did not reach statistical significance when compared to the TIV alone group (p = 0.12).

Overall, there was a significant negative correlation between the 7dpv plasmablast frequency and subject age (r = -0.46, p = 0.02) (Fig 1E), consistent with previous reports of reduced B-cell responses to influenza immunization in elderly human subjects [11].

**Fig 2:**
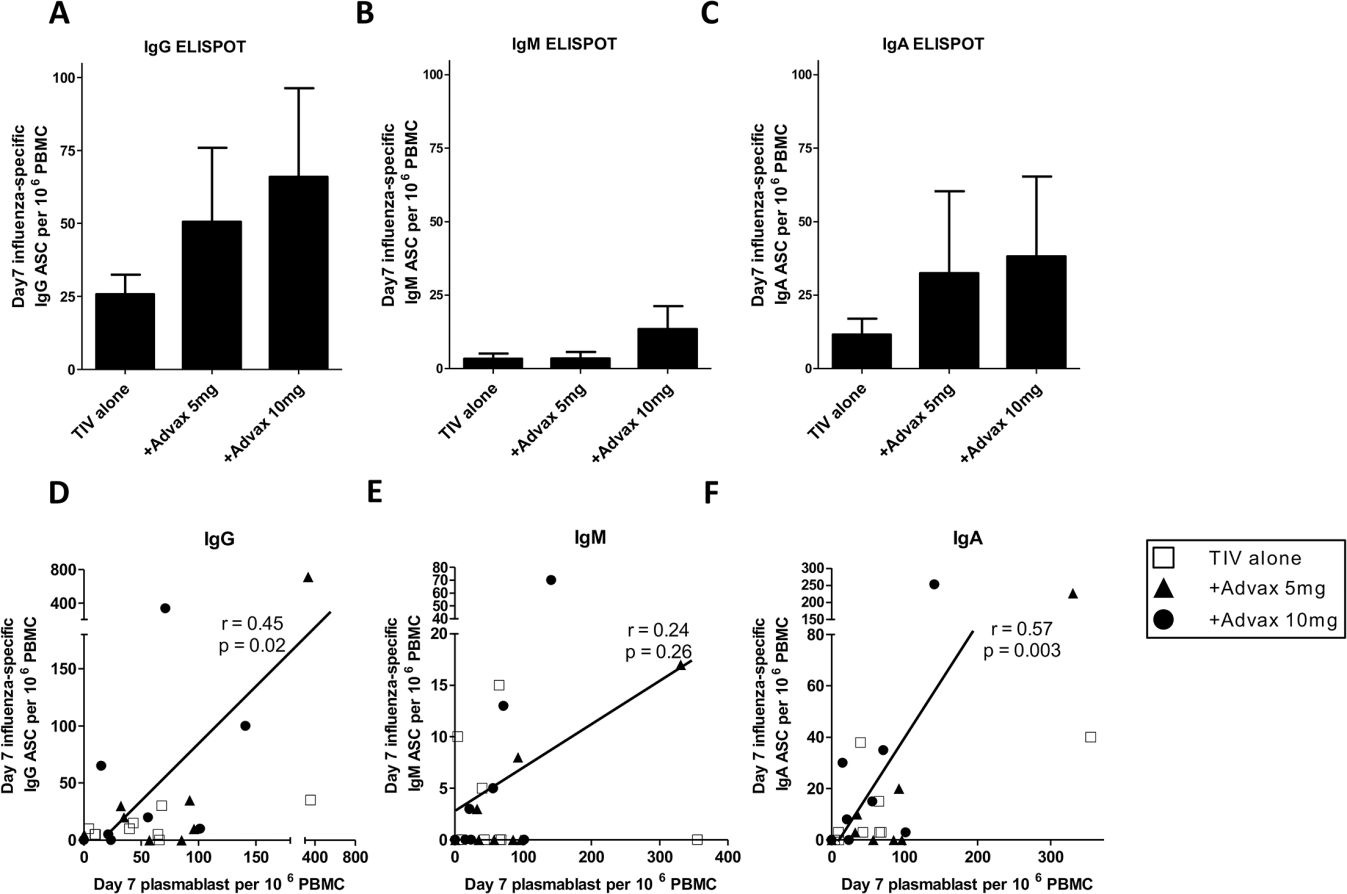
Effect of Advax adjuvant on post-immunization plasmablast response. The frequency of 7dpv TIV-specific B cells secreting IgG, IgM or IgA (mean ± SEM) was enumerated by ELISPOT (A-C). Plots show the correlation between plasmablast frequency 7pdv (measured by FACS) and influenza-specific IgG+, IgM+ or IgA+ plasmablasts 7dpv (measured by ELISPOT) (D-F).

Plasmablasts generated by influenza immunization have previously been shown to represent ~50% influenza-specific B cells. To confirm differences in plasmablast frequency represented true differences in influenza-specific B cell frequency, we undertook ELISPOT assays where thawed PBMC were placed directly into TIV-coated ELISPOT plates and cultured overnight (Fig 2). This assay detects plasmablasts spontaneously secreting large quantities of influenza-specific antibody, as confirmed by the absence of spots when using PBMC obtained pre-immunization or 28dpv when plasmablasts are absent in the peripheral blood (data not shown). There was considerable inter-individual variation in plasmablast frequency when measured by ELISPOT such that intergroup differences were not significant, which likely reflects the variable plasmablast viability following cryopreservation. Nevertheless, there were consistent trends that matched the FACS-generated plasmablast frequency data, with the highest frequency of influenza-specific IgG+, IgM+ and IgA+ spots in the blood of subjects that received Advax adjuvant (Fig 2A–2C). Overall, the highest percentage of influenza-specific plasmablasts secreted IgG, followed by IgA and then IgM (Fig 2A–2C). Point estimates of plasmablast frequency for each immunoglobulin isotype were highest in the Advax 10mg group.

When all groups were pooled for analysis, there was a significant positive correlation between the 7dpv-plasmablast frequency by FACS and 7dpv-influenza-specific plasmablasts detected by ELISPOT to be secreting IgG (r = 0.45, p = 0.02) or IgA (r = 0.57, p = 0.003) but not IgM (r = 0.24, p = 0.26) (Fig 2D–2F). This suggested that the generation of influenza-specific IgG+ and IgA+ plasmablasts correlated best with the overall size of the plasmablast response. This did not reflect a greater sensitivity of IgM+ plasmablasts to death from cryopreservation as the frequency of IgM+ plasmablasts was comparable in fresh and frozen PBMC from the same subject (data not shown).

We next assessed the correlation between the 7dpv plasmablast frequency and each subject’s 28dpv HI titer increase. Whereas there were significant correlations between plasmablast frequency 7dpv, and HI increases 28dpv, in subjects that received either Advax 5mg or Advax 10mg, there was no correlation in subjects that received TIV alone (Table 3). This suggests there is a direct link in the Advax groups between the plasmablast frequency 7dpv and the serum HI titers 28dpv.

**Table 3:** Correlation of plasmablast frequency 7dpv and HI titer increase 28dpv.

	TIV		+Advax 5mg		+Advax 10mg	
	Pearson r	p-value	Pearson r	p-value	Pearson r	p-value
**A/Cal**	-0.024	0.93	**0.65**	**0.0046	0.26	0.38
**A/Per**	0.20	0.45	0.32	0.22	**0.72**	**0.0034
**B/Bris**	0.37	0.16	**0.91**	***0.0001	**0.71**	**0.0046

Shown p-values represent the significance of the correlation between 7dpv-plasmablast frequency and 28dpv-HI titer increase within each group for each of the three vaccine antigens. No correction was made for multiple comparisons.

### Plasmablast BCR Affinity Maturation

**Fig 3:**
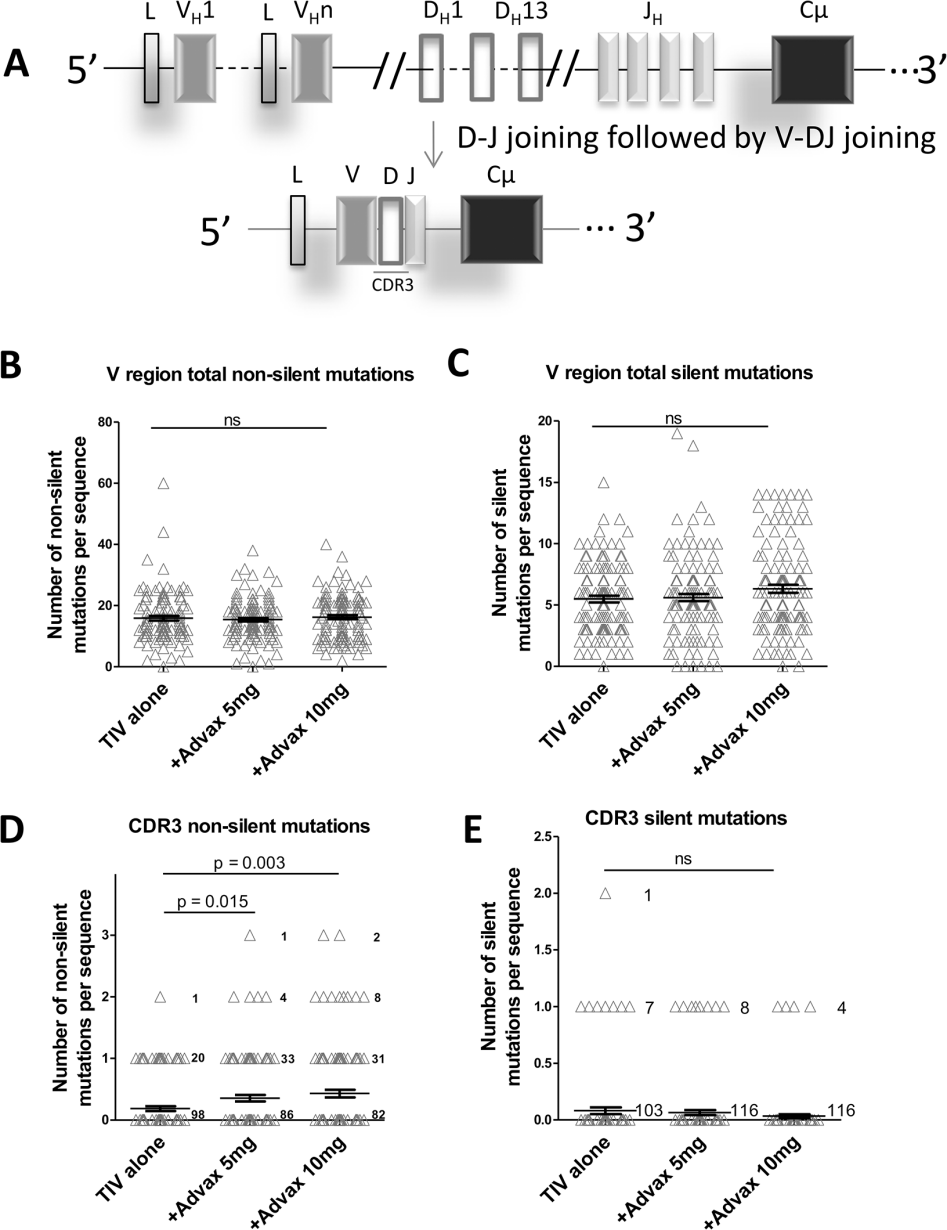
Advax adjuvant is associated with enhanced BCR heavy chain CDR3 affinity maturation. Schematic of human *Igh* gene and VDJ recombination (A). The IgG variable region cloned from sorted 7dp plasmablasts from each subject were analysed with IMGT/HighV-Quest online tool for mutation rate analysis. A minimum of 20 IgG variable region clones was sequenced from each subject. Shown is the frequency of non-silent (B) or silent (C) V region mutations and non-silent (D) or silent (E) CDR3-specific mutations.

To explain the higher neutralizing antibody titers in the Advax-immunized groups in previous influenza [9] and hepatitis B [10] vaccine studies, we hypothesized that Advax adjuvant might positively influence B-cell affinity maturation. Affinity maturation is a process whereby mutations are progressively introduced into the B-cell receptor (BCR) of proliferating antigen-specific B cells, allowing positive selection of the subset of daughter B cells expressing the highest affinity BCR for the antigen [17]. BCR antigen specificity is dictated by the complementarity determining regions (CDR), particularly the CDR3 formed by contributions from the V, D, J gene segments during BCR recombination, as depicted in Fig 3A. During B-cell affinity maturation, amino acid substitutions, additions and deletions are introduced into the CDR3 region thereby allowing generation of new non-germline BCR sequences with higher antigen affinity [18]. We hypothesized that if Advax was enhancing BCR maturation, this should be reflected by an increased frequency of plasmablast BCR amino acid substitutions focused in the antigen-binding CDR3 domain of plasmablasts in the Advax adjuvant groups. Plasmablasts were individually sorted 7dpv and BCR heavy chain genes from each subject cloned into individual expression libraries. Individual clones from each subject’s BCR heavy chain expression library were selected, sequenced and analyzed for mutations using the IMGT online tool [19]. The total frequency of BCR heavy chain mutations was not significantly different in 7dpv plasmablasts from subjects that received TIV alone (15.9 mutations/BCR sequence), TIV+Advax 5mg (15.4 mutations/BCR sequence) or TIV+Advax 10mg (16.2 mutations/BCR sequence). This was true whether the data was analyzed for frequency of non-silent mutations (inducing a change in amino acid sequence) (Fig 3B), or silent mutations (no change in amino acid sequence) (Fig 3C). This suggested the absence of any generalized effect of Advax on BCR mutagenesis.

**Fig 4:**
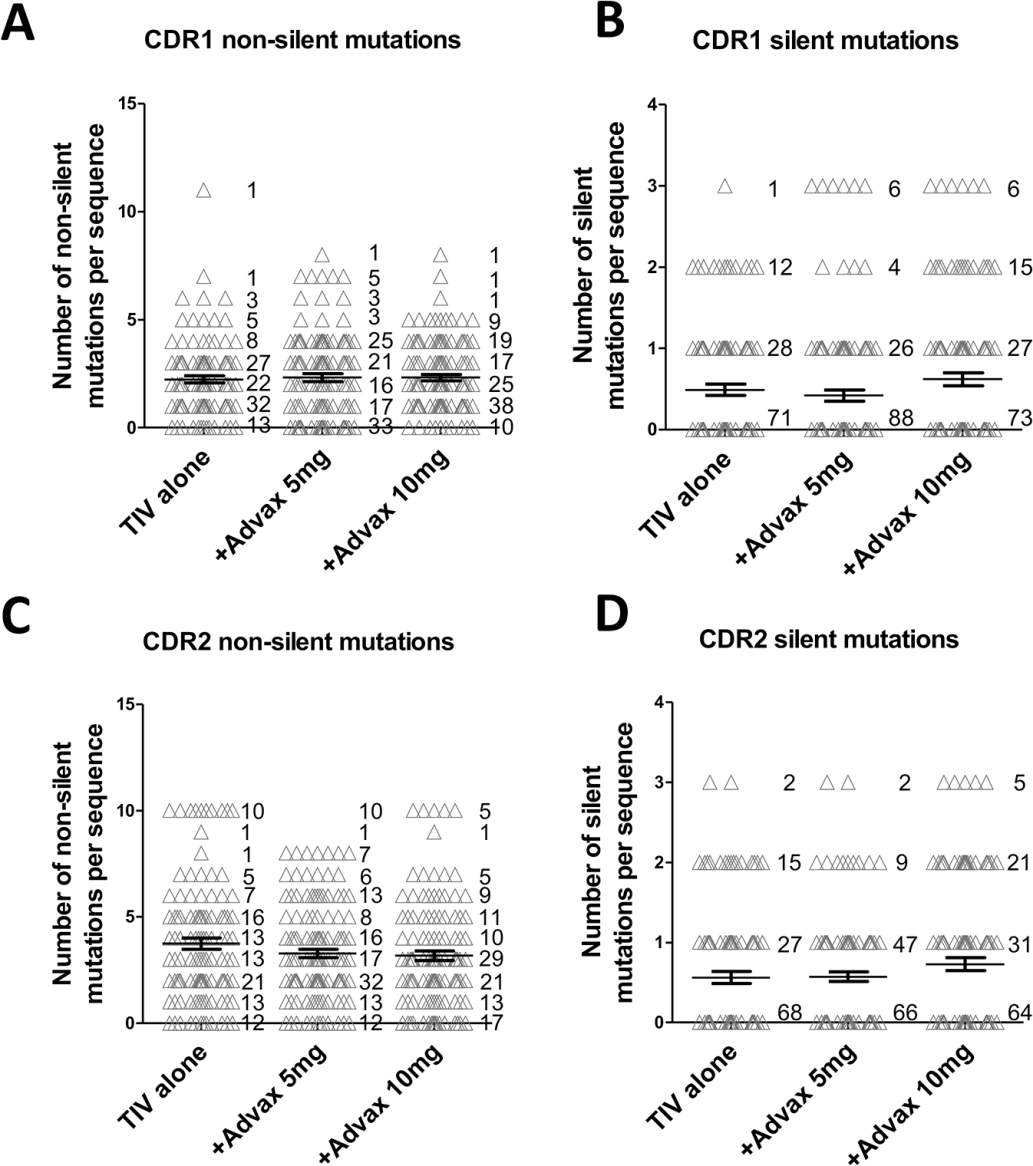
Advax adjuvant has no effect on CDR1 and CDR2 mutations. The IgG variable region cloned from sorted 7dpv plasmablasts from each subject were analysed with IMGT/HighV-Quest online tool for mutation rate analysis. A minimum of 20 IgG variable region clones was sequenced from each subject immunized with TIV alone (9 subjects), Advax 5mg (8 subjects) and Advax 10mg (7 subjects) subjects. Plots show non-silent and silent mutations for CDR1 (A-B) and CDR2 (C-D).

Next we restricted the mutation analysis to just the BCR CDR3 region, which is the main site of antibody rearrangement responsible for antigen-specificity [20]. Notably, there was a significant 2–3 fold higher rate of non-silent CDR3 mutations in 7dpv plasmablasts from the Advax 5mg group (mean = 0.35, SD = 0.05, p = 0.015) and Advax 10mg group (mean = 0.43, SD = 0.06, p = 0.003) when compared to the TIV alone group (mean = 0.18, SD = 0.04) (Fig 3D). As only non-silent CDR3 mutations result in a change in BCR sequence and are thereby under selective pressure as part of B-cell affinity maturation, we also compared the frequency of silent CDR3 mutations as a control. There was no significant difference between groups in the frequency of silent CDR3 mutations (Fig 3E) consistent with immunization with Advax being specifically associated with increased non-silent CDR3 mutations. Analysis of the CDR3 amino acid composition pattern of each vaccine group showed a clear trend to the Advax 10mg group having more variability of amino acid usage across positions 15–23 in the middle of the CDR3 region when compared to the TIV alone group (S1 Fig). Interestingly, there was no effect of Advax adjuvant on the rate of non-silent or silent mutations in CDR1 (Fig 4A–4B) or CDR2 (Fig 4C–4D), or on overall CDR3 length: TIV alone group (average CDR3 length 14.3aa, SD = 3.8); Advax 5mg group (14.6aa, SD = 3.3); and Advax 10mg group (13.8aa, SD = 4.2) (S2 Fig). Details of individual V region amino acid and sequences mutations are attached (S1 Table).

### Advax Enhances Plasmablast Activation-Induced Cytidine Deaminase (AID) Expression

**Fig 5:**
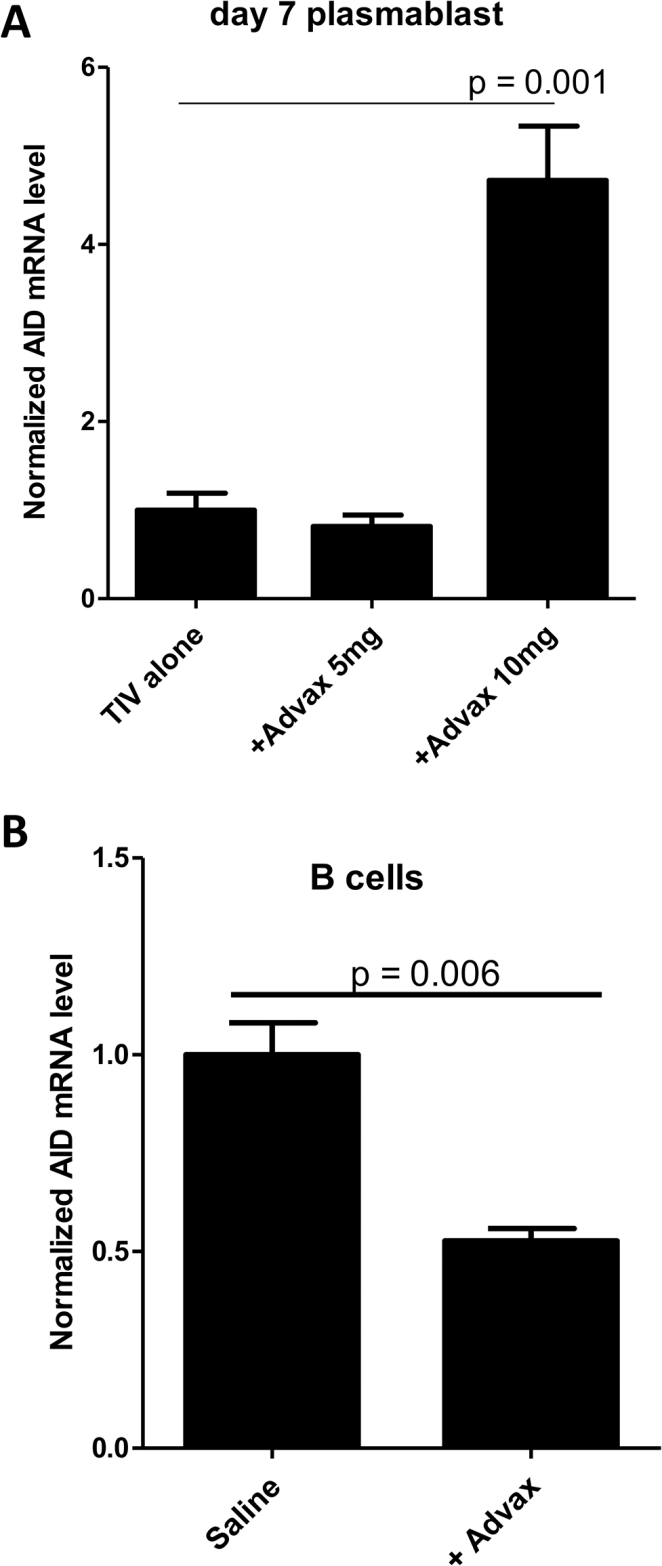
Advax adjuvant enhances plasmablast AID expression. Each subject’s 7dpv-plasmablasts were FACS-sorted into three pools per study group for RNA extraction and qPCR analysis of *AID* expression (A). To assess for a direct effect of Advax on B cells, PBMC were cultured with Advax adjuvant at 10μg/ml for 24h *in vitro* and then AID mRNA expression quantified by qPCR (B).

The finding that 7dpv plasmablasts in the Advax groups had a 2–3 fold increased rate of non-silent CDR3 mutations raised the possibility that Advax might be having an effect on BCR recombination, an essential process underlying B-cell affinity maturation. AID is a gene that encodes a DNA-editing deaminase that is involved in somatic hypermutation, gene conversion, and class-switch recombination of immunoglobulin genes [21]. We asked, therefore, whether subjects in the Advax groups had changes in plasmablast AID gene expression. Notably, plasmablasts from the Advax 10mg group had a highly significant ~5-fold upregulation of AID expression (p = 0.001) when compared to the TIV alone group (Fig 5A). Despite having a significantly higher rate of CDR3 mutations when compared to the TIV alone group, AID expression was not significantly increased in the Advax 5mg group.

To assess whether Advax might have a direct effect on B-cell AID expression, freshly isolated human PBMC were cultured with or without Advax then measured for AID gene expression by real-time PCR. Surprisingly, incubation with Advax significantly down-regulated AID expression in human PBMC (Fig 5B), suggesting the observed AID upregulation in 7dpv-plasmablasts from the Advax 10mg immunized group was not being mediated by a direct effect of Advax on B cells.

### Changes in Serum Antibody Avidity Post-immunization

**Fig 6:**
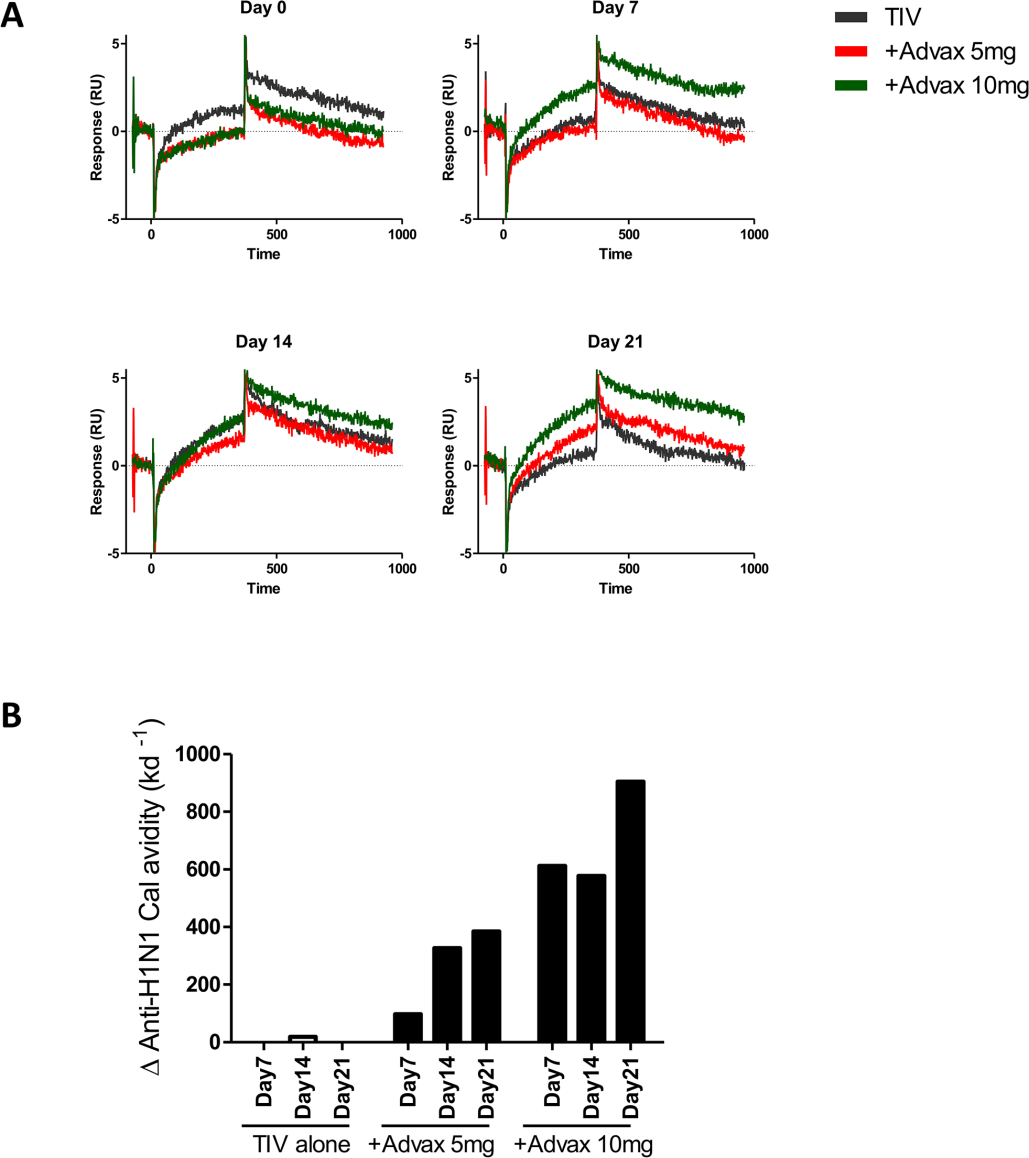
Advax adjuvant enhances influenza-specific IgG avidity. Surface plasmon resonance (SPR) was used to determine dissociation rate constants of influenza-specific IgG in pooled plasma from groups immunized with TIV alone, TIV+Advax 5mg or TIV+Advax 10mg. Shown are SPR sensorgrams of A/California/07/2009 antigen binding to captured IgG from serum pools from each group (A). Change from baseline in serum antibody avidity is presented as kd^-1^ (B).

To test whether the increased plasmablast CDR3 mutations in the Advax groups was associated with increased antibody avidity, surface plasmon resonance was performed on pooled 28dpv serum for each group for each time point post-immunization to assess binding and dissociation to A/California/07/2009 antigen, the H1N1 component of the 2012 TIV vaccine. Whereas post-immunization there was a clear step-wise increase in the quantity and avidity of H1N1-specific IgG in the pooled sera of each Advax group, there was no significant increase in IgG avidity in the TIV alone group (Fig 6) (S2 Table).

## Discussion

While Advax adjuvant when added to appropriate vaccine antigens has been repeatedly shown to enhance humoral and cellular immunity and thereby vaccine protection in animal models [6–8,22–24] and human trials [9,10], the mechanism for this remains unknown. This study used cryopreserved PBMC samples from a seasonal influenza vaccine trial to investigate how Advax adjuvant might be enhancing the human humoral response to influenza immunization at the cellular level. The results provide intriguing insights into the action of this unique polysaccharide adjuvant. As hypothesized based on previous animal studies where delta inulin adjuvant enhanced generation of antigen-specific memory B cells and plasma cells [6,25], administration to human subjects of influenza vaccine formulated with Advax adjuvant resulted in an increased plasmablast peak post-immunization, although surprisingly this effect was more evident at the lower Advax 5mg dose. This result also accords with a report findings that influenza immunization with an oil emulsion adjuvant increased the peak plasmablast response in human subjects [26]. The finding that Advax adjuvant was associated with an increased peak plasmablast response post-immunization, may at least partially explain the finding of higher antibody titers in human subjects receiving Advax-adjuvanted influenza vaccine the modest effect size on plasmablast frequency, particularly at the higher Advax 10 mg dose, suggested that some other mechanism of humoral enhancement might also be operating. To address the possibility that Advax adjuvant might also be having a positive effect on B cell affinity maturation, we compared BCR heavy chain usage in plasmablasts sorted from subjects that had received Advax versus TIV alone. Notably, plasmablasts from the Advax 10mg and 5mg groups both had significantly higher frequencies of non-silent BCR heavy chain mutations focused exclusively in the CDR3 region when compared to plasmablasts of subjects that received TIV alone. Plasmablasts from the Advax 10mg group, which had the highest rate of CDR3 mutations, also exhibited increased AID expression, consistent with the higher dose of Advax adjuvant having a positive effect on B-cell affinity maturation via upregulation of plasmablast AID. The diversity of the antibody repertoire as determined by the composition of the heavy chain CDR3 has been shown to be important to development of influenza heterosubtypic immunity [27], raising the possibility that the increased CDR3 diversity seen here in human subjects receiving Advax adjuvant might translate into enhanced influenza cross-protection. Whilst this possibility is yet to be tested in human subjects, a positive effect of Advax adjuvant on BCR CDR3 diversity might help explain the enhanced cross-protection against West Nile virus seen in mice immunized with Advax-adjuvanted Japanese encephalitis antigen, protection that could be transferred to naïve mice using memory B cells from immunized mice [28].

We next looked for a potential mechanism whereby Advax might be directly or indirectly be regulating B-cell function. TLR agonists such as CpG oligonucleotide and monophosphoryl lipid A are known to directly stimulate human B cell proliferation and antibody secretion via binding to receptors on the B cell surface [29,30]. Interestingly, this did not appear to be the mechanism of action of Advax as direct co-incubation with human B cells down- rather than up-regulated AID expression and had no effect on B-cell proliferation or antibody secretion (data not shown), suggesting that Advax actually has a direct inhibitory effect on human B cells. This suggests that the positive effect of Advax on AID expression and plasmablast generation is indirect and may therefore, for example, reflect an effect of Advax on T-follicular helper cell function that secondarily enhances B-cell AID expression and affinity maturation [31]. However, as we have been unable to show any direct interaction between Advax and human T cells (unpublished data), we hypothesize that any Advax effect on T cells is itself an indirect effect, such that the primary effect of Advax is likely to be on antigen presenting cells (APC) [5]. Under this model, Advax interacts with APCs leading to enhanced T-cell co-stimulation, these activated T cells provide help to antigen-specific B cells, resulted in enhanced B cell activation, proliferation, AID expression and affinity maturation, thereby explaining the enhanced production of high avidity serum antibody seen in subjects receiving Advax-adjuvanted vaccine.

Overall, the Advax dose was associated with the best overall vaccine response, as indicated by serum HI titers and antibody avidity, plasmablast AID expression and BCR non-silent heavy chain CDR3 mutations. The one exception was that plasmablast frequency post-immunisation was highest in the Advax 5mg group. This might suggest that plasmablast proliferation and AID expression are independently regulated with Advax 5mg primarily driving the former, and Advax 10mg, the latter. The Advax 10mg group was uniquely associated with an increased frequency of IgM+ plasmablasts 7dpv, a finding of unknown significance. Humans have a population of IgM+ memory B cells carrying a mutated immunoglobulin receptor that are proposed to correspond to circulating splenic marginal zone B cells [32]. In murine models, IgM+ memory B cells were shown to represent a long-lived precursor memory population that repopulates germinal centers and contributes to generation of high affinity plasmablasts and IgM+ and IgG+ memory B cells upon a secondary antigen exposure [33]. By contrast, IgG+ memory B cells only give rise to plasma cells. Hence, enhanced generation of IgM+ memory B cells by Advax might explain its ability in animal models to enhance heterologous virus protection [28].

A limitation of the current study as with other studies of human plasmablast function is the relatively low number of study subjects able to be studied, a reflection of the intensive nature of human cellular immune studies. Nevertheless, the key findings showing Advax adjuvant enhanced plasmablast frequency, AID gene expression and heavy chain BCR CDR3 mutations were all statistically significant. We are currently planning a larger human vaccine study to confirm and extend the current findings. Intensive studies are also underway in animal models, which are more amenable to experimental manipulation, to better understand how Advax might achieves these beneficial effects on B cell immunity. Ultimately this should provide much sought answers on how this novel polysaccharide adjuvant enhances vaccine protection while avoiding the toxicity of other adjuvants.

## Supporting Information

S1 Fig CDR3 amino acid composition pattern. Alignment of CDR3 amino acid sequences of individual BCR library clones derived from sorted 7dpv plasmablasts from individual subjects in each vaccine group was performed with ClustalW2 (http://www.ebi.ac.uk/Tools/msa/clustalw2/) and the pattern was plotted with WebLogo (http://weblogo.berkeley.edu/).
(TIF)

S2 Fig CDR3 amino acid length. Heavy chain CDR3 lengths of individual BCR library clones derived from sorted 7dpv plasmablasts from individual subjects in each vaccine group.
(TIF)

S1 Protocol Details of design of FLU006 clinical trial. (PDF)

S1 Table Details of individual BCR V region mutations and amino acid sequences from sequenced plasmablast BCR heavy chain libraries. (XLSX)

S2 Table Post-immunisation changes in influenza antibody avidity. Shown are Ka and Kd from surface plasmon resonance curve fitting of binding of inactivated A/California/07/2009 antigen to immune IgG captured onto Biacore chips. Chips were loaded with pools of patient sera representative of each vaccine group.
(XLSX)
