# Supplementary figures and images for: Delta Inulin Adjuvant Enhances Plasmablast Generation, Expression of Activation-Induced Cytidine Deaminase and B-Cell Affinity Maturation in Human Subjects Receiving Seasonal Influenza Vaccine
Source: PLoS One. 2015 Jul 15;10(7):e0132003. doi: 10.1371/journal.pone.0132003 (PMC4503308; doi:10.1371/journal.pone.0132003)

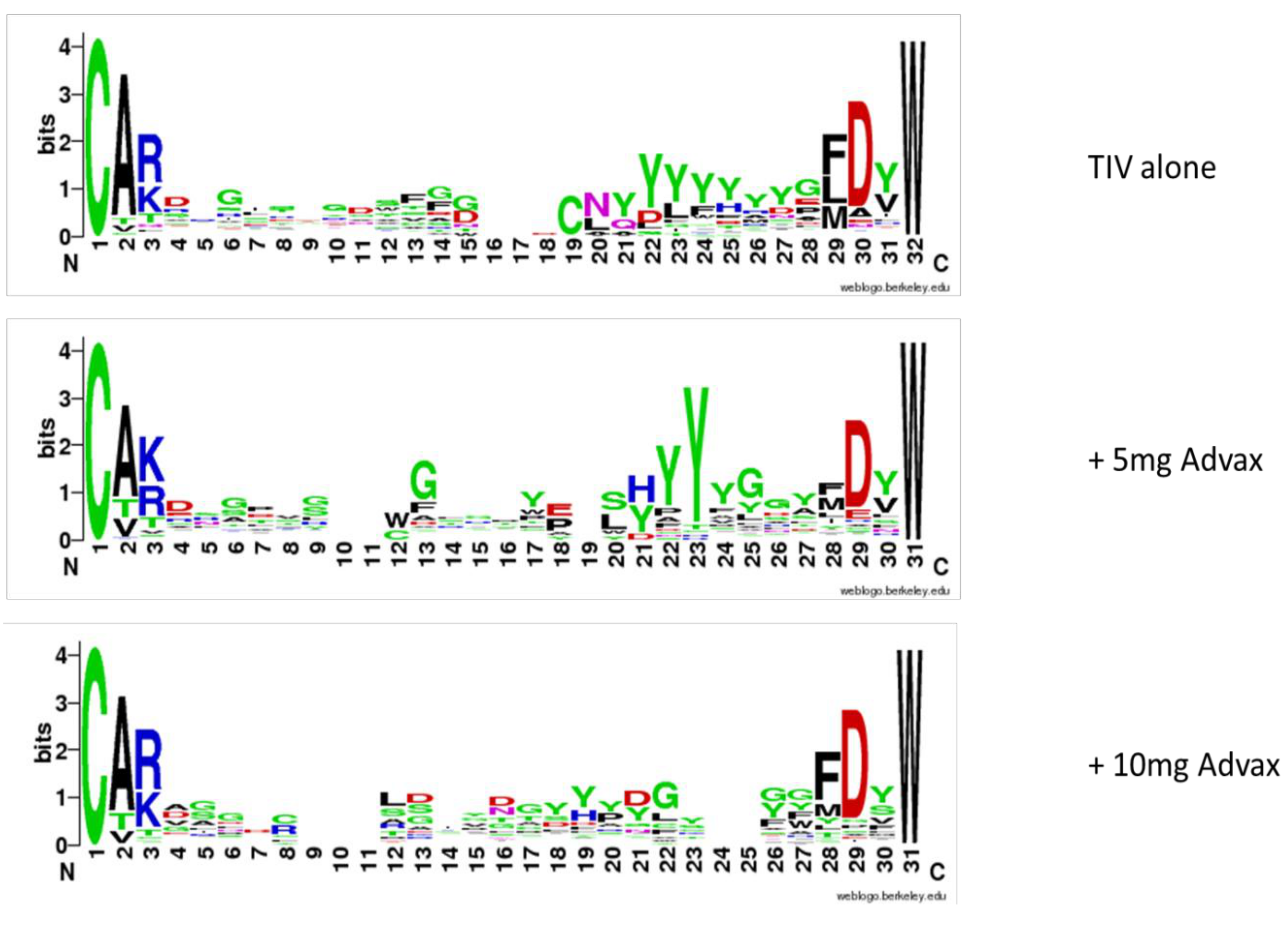

Supplement: S1 Fig — Alignment of CDR3 amino acid sequences of individual BCR library clones derived from sorted 7dpv plasmablasts from individual subjects in each vaccine group was performed with ClustalW2 (http://www.ebi.ac.uk/Tools/msa/clustalw2/) and the pattern was plotted with WebLogo (http://weblogo.berkeley.edu/). (TIF) [file pone.0132003.s001.tif]

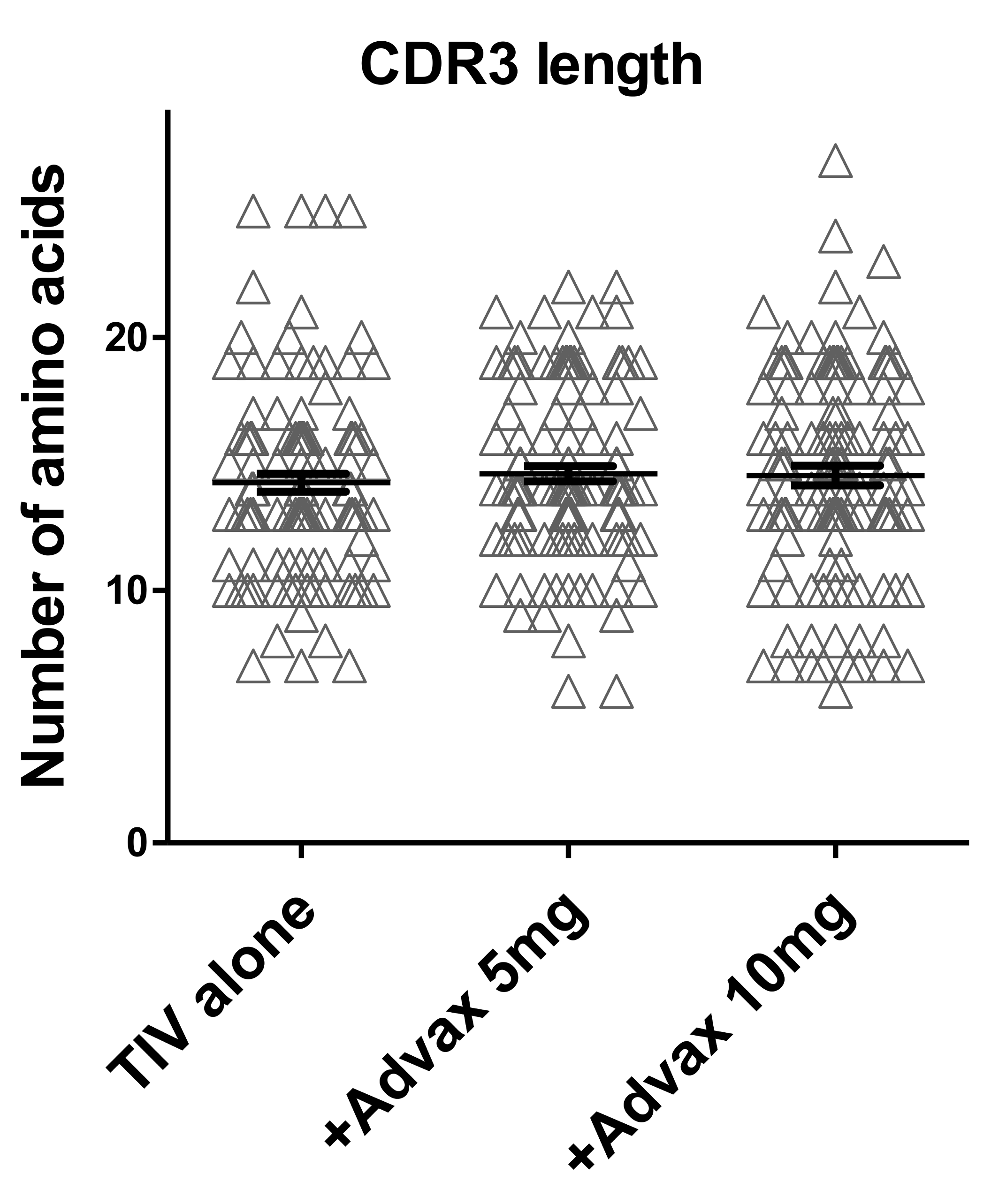

Supplement: S2 Fig — Heavy chain CDR3 lengths of individual BCR library clones derived from sorted 7dpv plasmablasts from individual subjects in each vaccine group. (TIF) [file pone.0132003.s002.tif]
