# Supplementary material for: Delta Inulin Adjuvant Enhances Plasmablast Generation, Expression of Activation-Induced Cytidine Deaminase and B-Cell Affinity Maturation in Human Subjects Receiving Seasonal Influenza Vaccine
Source: PLoS One. 2015 Jul 15;10(7):e0132003. doi: 10.1371/journal.pone.0132003 (PMC4503308; doi:10.1371/journal.pone.0132003)
Supplement: S1 Protocol — (PDF) [file pone.0132003.s003.pdf]

CLINICAL TRIAL PROTOCOL

TITLE:

A Randomised Controlled Trial of an Adjuvanted Seasonal Influenza Vaccine (FLU006)

SHORT TITLE: Adjuvanted Influenza Vaccine Study

STUDY CENTRE: Flinders Medical Centre, Bedford Park, Adelaide

PRINCIPAL INVESTIGATOR: Dr Dimitar Sajkov

STUDY PERIOD: March 2012 – December 2013

OBJECTIVES:

The primary objective is to assess the safety, tolerability and efficacy of an trivalent seasonal influenza vaccine formulated with Advax™ adjuvant by comparison to the trivalent seasonal influenza vaccine alone. A secondary objective is to assess the utility and patient acceptability of a needless vaccine injector (Pharmajet) by comparison to a standard intramuscular injection with syringe and needle.

Primary Endpoints:

- ***Efficacy:*** Seroconversion and seroprotection rates for each included serotype
- ***Safety:*** Adverse events and vaccine tolerability

Secondary Endpoints:

- Kinetics and persistence of seroconversion, seroprotection and B- and T-cell immunity for adjuvanted versus unadjuvanted influenza vaccine relative to adjuvant dose.
- Degree of cross-protection against drifted influenza strains for adjuvanted versus unadjuvanted influenza vaccine relative to adjuvant dose.
- Subject feedback and perceptions on needleless injector device

STUDY POPULATION:

Male and female subjects aged 18 years and older

FULL PROTOCOL

1. INTRODUCTION

1.1. Background

Influenza is a highly contagious and potentially deadly disease that can be spread through coughing or sneezing. It is caused by a virus and is not the same as a common cold. It can cause serious and debilitating complications such as pneumonia, especially in the elderly, young children and those with chronic disease.

The current study is designed to establish the safety and efficacy of the standard commercial trivalent inactivated influenza vaccine (TIV) when formulated with a range of adjuvant doses. We have shown in previous studies that addition of Advax™ adjuvant with various seasonal (TIV) or pandemic (H1N1/2009) influenza, or hepatitis B antigens improved the immunogenicity of these vaccines as assessed by higher antibody titres. This is the basis for extending study of Advax™-adjuvanted influenza vaccine to compare adjuvant doses lower to those previously tested and thereby identify the minimum adjuvant dose required for maximal vaccine enhancement.

Seasonal influenza vaccines are currently administered by intramuscular injection using a syringe and needle approach. New technology has made available a needleless injection system that is able to deliver the vaccine utilising a spring powered energy source to create a fine high velocity jet of liquid that directly penetrates through the skin without the use of a needle. The PharmaJet injector which is already approved for human use by the US Food and Drug Administration (FDA) employs a single-use, sterile, auto-disable needle-free cartridge which results in an superior injection experience for children and needle phobic subjects compared to use of needle-bearing syringes. This technology offers major advantages for both administrator and recipient over existing needle injection approaches and hence we plan to randomise trial subjects to receive their vaccine through either the Pharmajet device or needle and syringe, thereby allowing us to assess the influence on patient satisfaction and comfort of receiving their influenza vaccine through a needless device.

Adjuvants are a critical ingredient for many vaccines and act by boosting the immune response to vaccine antigens (e.g. influenza vaccine antigens). Despite considerable research, aluminium salts (alum) remain the dominant adjuvants used globally in human vaccines and there is a major need for new adjuvants, in particular, for adjuvants capable of boosting cell-mediated immunity (this is a particular type of immune response where T cells are activated that are then able to attack and destroy the infection). Alum, although good at stimulating antibodies, is poor at stimulating cell-mediated immunity. The majority of current commercial subunit flu vaccines, including those marketed in Australia do not contain an adjuvant.

FLUAD is a subunit influenza vaccine marketed by Novartis in Europe which contains a squalene oil adjuvant known as MF59. MF59 has been shown in clinical trials to induce higher influenza antibody titres when combined with seasonal or pandemic vaccines, albeit at the cost of higher local and systemic reactogenicity as reflected in

increased injection site pain, headaches and fever. Clinical data accrued to date suggests that Advax™ adjuvant may deliver similar benefits to MF59 adjuvant but without the propensity of MF59 to cause adverse reactions.

Advax™ adjuvant is manufactured under GMP from inulin, a natural storage polysaccharide of plants such as chicory and dahlias. Inulin in its soluble form is already approved in many countries for human intravenous use for renal function testing and is known to be an extremely safe and nontoxic compound. When crystallised, inulin develops properties including an ability to enhance the immunogenicity of co-administered vaccine antigens, which forms the basis for its use as a vaccine adjuvant. Previous clinical studies performed at FMC have confirmed the effectiveness and safety of Advax™ adjuvant when formulated with hepatitis B surface antigen, seasonal or pandemic influenza antigens or bee venom antigen. These studies all support the safety, tolerability and efficacy of Advax™ adjuvant in humans.

The 2012 seasonal influenza vaccine that will form the basis of this study is approved by the TGA. In this study, Advax™ adjuvant at either 5mg or 10mg will be formulated with the 2012 seasonal influenza vaccine, to allow us to confirm whether the reduced adjuvant dose delivers an equivalent anti-influenza antibody response. The rationale for testing a lower adjuvant dose is supported by data from a previous trial of a hepatitis B vaccine where Advax™ adjuvant at 5mg and 10mg doses was shown to be effective. If successful, this study would allow us to minimise the adjuvant dose and thereby maximise adjuvant availability, which could otherwise be a limiting factor, for example in an influenza pandemic where speed of production is critical.

1.2. Study Rationale

1.2.1 Overall study rationale

Extensive animal and human data suggests that addition of Advax™ adjuvant to standard influenza vaccines will enhance influenza immunity. This would be of particular benefit to those populations at high risk for influenza morbidity or mortality by providing them with higher levels of protection. Neutralising influenza antibody levels are accepted by the TGA as indicative of the level of vaccine protection.

1.2.2 Rationale for schedule and route of administration

The goal for influenza vaccination is to achieve a protective immune response in a minimum time frame and with the simplest dosing schedule. Animal data using several different models show that there is an increase in both the magnitude of the antibody response as the dose of antigen and adjuvant is increased to a critical levels but beyond this level the antibody response plateaus whilst the risk of adverse reactions continues to rise in a dose dependent manner. Hence it is critical to perform dose ranging studies to identify this “sweet spot” in the antigen and adjuvant dose response. Whilst previous clinical studies have compared different antigen doses, a fixed 20mg dose of Advax™ adjuvant was used. Hence there remains a need to be addressed in this study to now test different doses of adjuvant while keeping the dose of antigen fixed.

New technology has made available a needleless injection that delivers the vaccine through an intramuscular route without the use of a needle, instead utilising a spring powered energy source to inject liquid directly through the skin. The PharmaJet injector

employs a single-use, sterile, auto-disable needle-free syringe that results in an arguably superior injection experience and sensation compared to needle-bearing syringes. This device is FDA approved. Based on the above, we will trial a one dose regime of seasonal influenza vaccine delivered using standard needle and syringe or the PharmaJet needleless injector in a 1:1 ratio for all groups. This will enable us to assess patient satisfaction when using a needless injector compared to needle-bearing vaccination administration.

1.2.3 Rationale for selection of doses

Currently available commercial seasonal influenza vaccines contain 15 micrograms of influenza haemagglutinin (HA) from the three influenza virus serotypes (two A serotypes and one B serotype) approved by TGA for that particular year's vaccine. This equates to a total of 45 micrograms of HA per vaccine in 0.5mls volume. As the needless injector has a capacity of 0.5mls this means that it can inject the full dose of influenza HA. When formulated with Advax for use in the needleless injector, this will reduce the dose of influenza HA by approximately 20% in order to keep the volume to 0.5mls. However, we have previously shown that much greater HA dose reductions (3-10 fold) had little impact on the immunogenicity of influenza vaccine when combined with Advax™ adjuvant.

We have previously demonstrated in other clinical studies that Advax™ adjuvant is safe and effective in the dose range of 5 to 20mg although only the 20mg dose has been tested with influenza vaccines. In this study, we wish to test whether the same benefits can be achieved from a lower 5mg or 10mg dose of Advax™. This would reduce the dose of adjuvant required for each vaccine and thereby provide benefits in terms of reducing the cost of each vaccine and increasing adjuvant availability, for example in a pandemic, where a large number of vaccine doses might be required in a short space of time

2. STUDY OBJECTIVES

2.1 Primary Endpoints:

- Seroconversion and seroprotection rates and mean GMT increases in haemagglutination inhibition titres 21-28 days post-immunisation for each of the three included vaccine strains

2.2 Secondary Endpoints:

- Pain scores and subject satisfaction with needleless injection device
- Anti-influenza T-cell responses and kinetics
- Persistence of anti-HA antibody levels and T-cell immunity to influenza
- Cross-protection against drifted influenza strains
- Study of immune cell gene correlates of vaccine immunogenicity and reactogenicity
- Influenza plasmablast frequency at day 7 post-immunization

3. STUDY SITES

The study will be conducted at Flinders Medical Centre (FMC, Adelaide, South Australia) with the option to add additional study sites as required to reach recruitment targets.

4. STUDY DESIGN

This is a Phase 2, randomised, controlled trial of the comparative efficacy of either of two doses of Advax™ adjuvant when combined with 2012 seasonal influenza vaccine as administered by either standard intramuscular injection or a needless injector device

4.1.1. Schedule of study visits

Day 0 - Visit 1 **(All subjects)**
After providing informed consent, and being assessed for eligibility criteria, study subjects will have a medical history taken, baseline clinical assessment, and blood taken for measurement of baseline titres of anti-influenza antibodies. Each eligible subject will then be randomised and receive vaccination with one of the study vaccines. Subjects will be observed for 30 minutes following immunisation and will then be provided with a subject diary to take home with them to record any symptoms or adverse events

Day 1 – By telephone (optional)
Telephone visit to assess subject reaction to vaccine

Day 7
Visit to provide blood samples specifically for measurement of influenza plasmablast responses

Days 14 and 21- **Substudy subjects only**
Visit to provide blood samples specifically for measurement of B- and T-cell responses. This visit will only involve the subset of subjects who specifically consent to be in the substudy that will involve weekly attendance for 4 weeks for collection of blood.

Day 28 – Final Visit **(All subjects)**
Final study visit to provide blood samples and for collection of patient diary. Subjects will have their injection site examined and have any adverse events recorded.

Day 185-365 – By telephone (optional)
Telephone visit to ascertain subject wellbeing

5. ANALYSIS

5.1 <u>Evaluation of vaccine efficacy</u>

<u>(a) Immunogenicity tests</u>

1. Anti-influenza antibody titres
2. Anti-influenza B and T-cell responses

5.1 <u>Evaluation of vaccine tolerability and safety</u>

This analysis will utilise the FDA/CBER toxicity grading scale for healthy adult and adolescent volunteers enrolled in preventative vaccine clinical trials (www.fda.gov/cber/gdlns/toxvac.htm). Specified solicited adverse events will be

collected for 7 days post-immunization and unsolicited adverse events for the total 6 month follow up period.

5.1.2 Elicited Local symptoms/signs

- -pain, tenderness, erythema/redness, induration/swelling
- Vital signs
- -fever, tachycardia, bradycardia, hypertension (systolic/diastolic), hypotension, increased respiratory rate

5.1.3 Elicited Systemic (general) symptoms/signs

- Nausea/vomiting, diarrhoea, headache, fatigue, myalgia
- Interference with activity, need for medical attention, ER visit/hospitalisation

5.1.4 Physical examination

Local - inflammation, induration, redness, granuloma, skin breakdown
Systemic - Fever, tachycardia, hypertension

5.2 Choice of control groups and blinding

This is a randomized, controlled Phase 2 study. A total of up to 400 subjects will be recruited for the study, and randomly assigned on a one to one to one basis (1:1:1) to standard TIV, TIV + Advax 5mg or TIV + Advax 10mg. All subjects will also be randomised on a 1:1 basis to receive the immunisation via needle and syringe or Pharmajet needleless injector device. There will therefore be 6 subgroups in total.

As the vaccine containing Advax is a cloudy white colour whereas TIV is a clear solution, it is not possible for the staff administering the vaccine to be blinded. Subjects will be blinded and will not be informed which vaccine they receive. Similarly, where a trial adverse effect requires assessment, this will be performed by a blinded adjudicator. All laboratory tests will be performed blinded.

5.3 Study Visit Window

Scheduled Vaccination and Follow up Visits will be treated as within the study protocol, if performed plus or minus 7 days from the study day specified in the protocol. If a subject has a fever at a Vaccination Visit, then the Visit can be rescheduled by up to four weeks without recording a protocol violation.

5.4 Safety Monitoring Board

Given the short duration of this study and the fact that just a single immunization will be given on study day 0, a safety monitoring board (SMB) is not deemed suitable for this study. The Principal Investigator will monitor all serious adverse events and if considered to be related to study medication this will be notified to the ethics committee.

6. STUDY POPULATION

The study population will comprise up to 400 subjects 18 years and older, who fulfil the inclusion criteria, do not meet any of the exclusion criteria, and who have given informed consent.

6.1 Inclusion criteria

- Male or female
- Age 18 years or over
- Able to provide written informed consent
- Willing and able to comply with the protocol for the duration of the study.

6.2 Exclusion criteria

- History of vaccination with the 2012 seasonal influenza vaccine
- History of serious vaccine or egg allergy
- Women of childbearing potential, unless using a reliable and appropriate contraceptive method, specifically oral contraceptive pill, IUD or mechanical barrier device.
- Pregnant or lactating women.
- Receipt of another investigational agent within 14 days preceding initiation of treatment.
- Any other serious medical, social or mental condition which, in the opinion of the investigator, would be detrimental to the subjects or the study.

6.3 Replacement policy

Individuals dropping out of the study prior to Visit 3 will be replaced at the discretion of the Principal Investigator.

7. TREATMENTS

7.1 Treatments administered

Study subjects will receive the vaccine by intramuscular injection into the deltoid muscle using a needle and syringe or needleless injector device.

7.2 Identity of investigational product

The influenza vaccine will be formulated as either 2012 seasonal TIV or the same TIV formulated with 10mg or 20 mg Advax™ adjuvant.

7.3 Supply, packaging and labelling

All vaccines will be stored at 4°C. Each vial of study drug will have a pre-printed label including the following information: study number, bulk batch number, and volume. A space is also provided for the person administering the drug to enter the individual's ID number and initials at the time of administration. The vial will be labelled for Clinical Trial Use Only.

7.4 Preparation

A minimum of 30 minutes before administration the vaccine will be transferred from 4°C storage to room temperature for temperature equilibration. Standard TIV will be supplied in a pre-packaged syringe with needle as supplied by the manufacturer. For subjects receiving the adjuvanted vaccine, the adjuvant will be supplied and mixed with vaccine as per the vaccine administration SOP. The vaccine is mixed by repeated inversion before the complete contents of the vial are aspirated into either a new 1ml syringe or the needleless injector device disposable cartridge. The formulated vaccine can be kept at room temperature for 8 hours. Before administration, the adjuvanted vaccine syringe/cartridge should be inverted a number of times to ensure even mixing of

the adjuvant. Standard influenza vaccine is a clear solution whereas the vaccine containing adjuvant is a cloudy white colour.

7.5 Administration of injection
The total dose will be given as a single injection into or over the deltoid muscle using a suitable disposable sterile syringe mounted with a 25-27G needle if in the standard syringe adjuvanted TIV groups, pre-packaged syringe and fixed needle if in the standard syringe TIV alone group, or the needleless applicator if in needleless injector groups.

7.6 Emergency Procedures
For vaccines the incidence of anaphylactic reactions are negligible, but emergency equipment must be available.

In case of anaphylactic shock the SA Health Anaphylaxis – Management Guidelines CC1.255 will be followed, as attached

Cases of anaphylactic shock must be reported as SAEs. Treatment should be permanently discontinued should such an event occur.

7.7 Storage, issue, return and accountability of study drugs

7.7.1 Storage
Study vaccines must be stored at a temperature of 4°C. Freezing may destroy the effectiveness of the vaccine. The study vaccines and adjuvant must be stored in a secure location at the investigator's site and may be dispensed only by the investigator or by a member of staff specifically authorised by the investigator.

7.7.2 Issue and return of study vaccines and adjuvant
The dispensing of the study vaccine to the investigators and subsequent administration to study subjects will be recorded on the drug accountability form and accurate accounting will be given to the monitor at each monitoring visit. Unused study vaccines must not be discarded or used for any purpose other than the present study.

7.7.3 Accountability of study vaccine
All vaccine supplies for this study should be retained in a safe secure place at all times during the study. The investigational product(s) should be administered by the investigator, or by a qualified individual under the investigator's supervision. An up to date vaccine inventory/dispensing record must be maintained. The individual's ID number and initials should be written on each vial just before it is administered to an individual. The empty or partially empty vials must then be stored, so that the vials administered to each individual can be verified by the study monitor. After completion of the study, all unused material must be accounted for. If the investigator or hospital pharmacist destroys the supplies, written documentation is mandatory. The record must include the following: who destroyed the supplies, the identity and quantity of what was destroyed, and the method and date of destruction. All drug supplies must be accounted for. At the end of the study, a copy of the vaccine inventory/dispensing record should be stored and a copy kept in the investigator's study file. The investigator is responsible for the vaccine supplies.

7.8 Criteria for dose modification, dose escalation or vaccine discontinuation

7.8.1 Dose modification

The dose administered should always be in accordance with the protocol. If a dose other than that shown in the protocol is administered then this should be recorded as a protocol violation.

7.8.2 Vaccine discontinuation

Individuals who have had past history of potential vaccine reactions will be assessed by the investigator, who will decide whether any past potential vaccine-related side are sufficiently minimal to allow the subsequent administration to proceed.

7.8.3 Dose limiting toxicities

Toxicities that must be considered as serious adverse events (SAEs) are:

1. Grade II to IV allergic reactions.
2. Grade IV neutropenia.
3. Grade IV thrombocytopenia.
4. Grade III to IV hypotension.
5. Grade III to IV dyspnea.
6. Occurrence of autoimmune disease.
7. Any Grade IV toxicity (except fever and flu-like symptoms which can be treated with paracetamol).
8. Recurring Grade III toxicities.

8. STUDY PROCEDURES AND EVALUATIONS

8.1 Assessment periods

One immunisation will be performed on Day 0 (Visit 1). Although the final study visit takes place 4 weeks after immunization, the total follow up period may be up to 12 months with a provision for a telephone call to the subject during this period to ascertain any new medical events that might have occurred during this interval.

8.2 Screening procedures

Only subjects who fulfil the inclusion and exclusion criteria, and for whom written informed consent has been obtained, will be eligible for enrolment into the study, and consent must be obtained before any screening procedures are undertaken. Screening of individuals should take place within 28 days prior to the start of treatment. At the time of screening the following assessments/procedures will be performed:

1. Inclusion and exclusion criteria.
2. Written informed consent.
3. Demographic data (sex, race, date of birth).
4. Pregnancy test (if applicable)
5. Medical history, vaccination history, concurrent illnesses including details of primary diagnosis and previous treatments.
6. Physical examination.
7. Vital signs (supine blood pressure, heart rate, temperature).
8. Body weight and height.
9. Blood sample for influenza antibody and T-cell assessment.

8.3 Randomisation

Study participants will be randomized to one of the treatment groups in the order of their enrolment in accordance with a predetermined balanced randomization protocol.

8.4 Prior to treatment procedures

The assessments that should be conducted at the visits are summarised in the Schedule of Assessments. Details of the evaluations are given below:

1. Physical evaluation.
2. History and concomitant medication.
3. Blood sample for baseline influenza antibody and T-cells before vaccine administration

8.5 Follow-up

The assessments that should be conducted at these visits are given in the Schedule of Assessments. The following evaluations will be performed.

1. Vital signs (blood pressure, heart rate, temperature).
2. Inspection of immunization site
3. Blood sample for influenza antibodies and T-cell responses and safety tests where indicated.
4. Adverse event history.

8.6 Early termination

At early termination of the study due to excess adverse events or any other reason the following procedures/tests will be performed.

1. Physical examination and vital signs (blood pressure, heart rate, temperature).
2. Blood sample for haematology and biochemistry (where indicated).
3. Blood sample for influenza antibodies and T-cell responses.
4. Recording of adverse events.

8.7 Observations and measurements: Assessment for treatment effects

8.7.1 Medical history

Information obtained will include history of underlying disease and concurrent illnesses (including the date of onset, if known), concomitant medication, vaccination history during the last 5 years before entry into the study and surgical procedures.

8.7.2 Laboratory assessments

Local and central laboratory analyses

Routine laboratory tests

Haematology and blood chemistry may be performed on selected subjects at discretion of the Investigator. Haematology assessment may include complete blood count including differential and platelet count, haemoglobin and haematocrit, ESR and coagulation studies. Biochemistry may include glucose, blood urea nitrogen, sodium, potassium, bicarbonate, chloride, creatinine, alkaline phosphatase, gamma-glutamyl transpepsidase (GGT), total bilirubin, aspartate transaminase (ASAT), alanine transaminase (ALAT), calcium, inorganic phosphorous, albumin, total protein and uric acid, rheumatoid factor and anti-nuclear antibodies, total haemolytic complement, C reactive protein, C3 and C4.

Immune response assays.
Blood samples will be drawn for the evaluation of influenza antibody titres of all the individuals immediately prior to immunization, and 7 days and 4 weeks post immunization. Those subjects who consent to be in the Substudy will have blood taken weekly for 4 weeks post-immunisation for analysis of B- and T-cell responses to influenza and control antigens.

9. STATISTICAL METHODOLOGY AND ANALYSIS

9.1 Evaluations

9.1.1 Primary variables

(a) Antibody responses to each vaccine serotype as assessed by TGA criteria
(b) Frequency and severity of SAE and AE including laboratory safety assessments.

9.1.2 Secondary variables

(a) Injection tolerability and acceptability scores by questionnaire and visual analogue scales
(b) Measurement of B and T-cell responses to influenza antigens
(c) Gene expression changes correlating with vaccine outcomes (immunogenicity and/or reactogenicity)

9.2. Description of individual groups for analysis

9.2.1 Safety population

All enrolled individuals who received at least one dose of treatment will be included in the safety population.

9.2.2 Per-protocol population (PPP)

All individuals who received at least one dose of treatment and who provided baseline and at least one post-baseline measurement for influenza antibodies will be included in the per-protocol population.

9.3 Statistical analysis

A descriptive analysis will be performed. All variables will be analysed descriptively. The safety analysis will be performed for the safety population. The efficacy analysis will be performed for the PPP population.
Protocol violations will be listed by patient. Protocol violations include the following:

(a) Non-compliance of the inclusion and exclusion criteria.
(b) Non-permitted concomitant medication
(c) Scheduled visit occurring outside of visit window

9.4 Efficacy analysis

All continuous data will be described by means of summary statistics: N, mean, standard deviation, minimum, median and maximum and 95% confidence intervals. Categorical variables will be presented using the number of observations (N), frequencies (%) and missing data.

The efficacy variables will be described by adequate methods in tables by visit and treatment group. The change from baseline to endpoint (if necessary defined as the last

value carried forward) will also be described by summary statistics. Influenza-specific antibody titres (IgM, IgG, IgG1-4, haemagglutinin inhibition (HI) titre) will be analysed by calculating changes from the background pre-immune serum readings.

All individuals with at least one post-baseline measurement for efficacy will be included in the efficacy population. The analysis will include listings of objective response according to currently pertaining TGA influenza vaccine criteria, (e.g. seroconversion = four fold increase in HI over baseline, seroprotection = post-immunisation HI of 1:40 or greater: potency = fold increase in HI geometric mean titres (GMT). GMT and 95% confidence intervals will be estimated pre- and post-immunisation and compared across groups by ANOVA. The proportion of each vaccine group achieving seroconversion or seroprotection will be compared by Fisher's exact test.

An efficacy analysis to identify differences in treatment response between unadjuvanted and adjuvanted TIV groups and between needle and needless injector groups will be performed on the complete PPP irrespective of age. Additional subgroup efficacy analyses to identify significant differences in treatment response between unadjuvanted and adjuvanted TIV groups and between needle and needless injector groups will be performed stratifying subjects by age.

9.5 Secondary Endpoints

Post-immunisation pain scores will be assessed by visual analogue pain charts which will be completed by study subjects immediately after immunisation and 30 minutes after immunisation. Pain scores at each time point will be compared across groups by ANOVA. Influenza antibody responses will be assessed by ELISA or neutralisation assays with drifted influenza strains to assess potential cross-protection. The proportion of each vaccine group achieving seroconversion or seroprotection against specific viral strains will be compared by Fisher's exact test. Changes from baseline to endpoint will be calculated for anti-influenza T-cell responses. Best overall response and duration of overall response will be summarized.

9.6 Safety analysis

All adverse events will be summarized and listed with a detailed description, using the MedDRA dictionary. Frequencies of adverse events (number of patients with the event, number of mentions of the event and percentage of patients exhibiting the event) for each vaccine formulation and immunisation route will be given for all adverse events. Adverse events will be summarized by vaccine formulation and immunisation route, body system and relationship to study drug and severity. In addition, listings will be prepared for adverse events leading to treatment discontinuation and for SAEs.

The vital signs (sitting blood pressure, heart rate and temperature) will be presented with summary statistics over the visits and for the change from baseline to endpoint. Individual laboratory variables over the visits and the change from baseline to endpoint will be listed with summary statistics for each dose level. The frequencies of laboratory values below, within and above the relevant normal ranges will be given. The baseline and endpoint laboratory variables will be presented using a contingency table to investigate any range changes. The abnormal and clinically significant laboratory variables will be presented for each patient over the assessments.

All enrolled individuals who received at least one dose of treatment will be included in the safety population. The analysis will include listings and frequency tables of adverse events and laboratory values graded according to the FDA/CBER vaccine toxicity grading scale. Adverse events will be tabulated by time (day) and by severity (mild, moderate and severe). Clinically significant reactions will be defined as measured local reactions >10mm, fever >38°C and severity $\geq$ moderate for other symptoms. Severe reactions are defined as measured local reactions >50mm, fever >39°C and severity $\geq$ severe for all other symptoms. Injection site reactions will be combined to give an "any local" reaction category and all other symptoms combined to give an "any general" reaction. The proportion of subjects having an adverse reaction will be estimated by vaccine group, observation period and severity. Subjects will be asked to score injection site pain at the time of injection, and 30 minutes post immunization using a visual analogue scale. Binomial distribution point estimates and 95% confidence intervals will be used to estimate each rate. Percentages will be compared by Fishers exact test. $P<0.05$ will be considered statistically significant. No adjustments will be made for multiple comparisons.

9.7 Sample size estimation

The study population will comprise up to 400 subjects 18 years and older, who fulfil the inclusion criteria, and do not meet any of the exclusion criteria who will be randomised into 6 groups of 65 subjects per group. The TGA based on long standing experience with influenza vaccines requires a minimum group size of 50 subjects per group for assessment of influenza vaccine antibody responses for vaccine release purposes. Therefore, rather than using a power calculation based on our own limited assumptions, we have instead relied upon this TGA requirement as our guide in designing the group size for this study, further allowing for a 20% drop out rate for subjects failing to attend for the final blood sample, thereby ensuring we meet the TGA minimum group size.

9.8 Subgroup analyses

A pooled efficacy analysis will be performed on the total study efficacy population. In addition, subgroup analyses of vaccine response rates will be performed stratifying responses according to the subject age and analysis of differences in GMT by ANOVA and seroconversion and seroprotection rates by Fishers exact test.

9.9 Data handling and quality assurance procedures

Accurate and reliable data collection will be assured by verification and cross-checking of the CRF against the investigator's records by the study monitor, and the maintenance of a drug dispensing log by the investigator. All correspondence between investigators and study monitors addressing apparent inconsistencies or to clarify entries on the CRF will be kept on file at the site. The data entry group will enter data from the working copy of the CRF (retrieved from the centre) into a computer database.

9.10 Randomisation procedure

Random allocation sequences will be generated by an independent source. . Randomisation codes will be held independently to the site. To randomise a subject the investigator or study co-ordinator will use an on-line interface to enter subject initials and date of birth. On entering this data, they will be issued with the next available randomisation code for the particular randomisation block or catagory to which the entered subject belongs, e.g. with or without Advax, 10mg or 20mg etc.

10. DRUG SAFETY

10.1 Definition of adverse events and SAEs

10.1.1 Adverse event

An adverse event is defined as:
Any untoward medical occurrence in an individual or clinical investigation subject who has been administered a pharmaceutical product and which does not necessarily require a causal relationship with the treatment. An adverse event can therefore be any unfavourable and unintended sign (including an abnormal laboratory finding, for example), symptom, or disease temporarily associated with the use of a medicinal product, whether or not considered related to the medicinal product.
Specifically, adverse events include:

1. All unfavourable changes in general condition.
2. All subjective or objective symptoms.
3. All concomitant diseases or accidents.
4. All clinically relevant changes in laboratory parameters.

Post-immunisation adverse events will be specifically defined as those adverse events occurring in the first 28 days post-immunisation. However, adverse event data will continue to be collected for the duration of the study follow-up period of 1 year post-immunisation

10.1.2 Serious adverse event

An SAE (or experience) or reaction is any untoward medicinal occurrence that at any dose:

1. Results in death.
2. Is life-threatening
3. Requires an individual's hospitalisation or prolongation of existing hospitalisation (over and above the period of hospitalisation foreseen in the protocol).
4. Results in persistent or significant disability/incapacity.
5. Is a congenital anomaly/birth defect.

(Note: the term "life-threatening" in the definition of "serious" refers to an event in which the individual was at risk of death at the time of the event; it does not refer to an event which hypothetically might have caused death if it were more severe).

The term "severe" is often used to describe the intensity (severity) of a specific event (as in mild, moderate or severe myocardial infarction); the event itself, however, may be of relatively minor medical significance (such as severe headache). This is not the same as "serious", which is based on individuals/event outcome or action criteria usually associated with events that pose a threat to an individual's life or functioning. Seriousness (not severity) serves as a guide for regulatory reporting obligations. Any death occurring during the study or which comes to the attention of the investigator within 4 weeks after stopping the treatment, whether considered treatment-related or not, must be reported. In addition, drug misuse or drug overdose should be regarded as an SAE, even if they may not result in the above mentioned outcomes. All SAEs must be reported immediately according to the guidelines for subsequent reporting to the appropriate regulatory authorities. The investigator must notify the Ethics Review

Committee of such an event in writing as soon as it is practical and in accordance with international and local laws and regulations.

10.1.3 Unexpected Adverse Event

An experience not previously reported (in nature, severity or incidence) in the current Investigator's Brochure as provided, is defined as an unexpected adverse event.

10.1.4 Methods of recording and assessing adverse events

All adverse events must be documented in the section of the CRF reserved for this purpose and not in remarks elsewhere in the CRF. The following aspects must be recorded

-The onset.
-Where applicable, the end of the adverse event.
-A description of the adverse event.
-Any factors considered as possibly promoting the occurrence of the adverse event.
-Concomitant medication.

An assessment by the investigator of the causality; the decisive factor in the documentation is the temporal relationship between the adverse event and the study drugs. The following judgments of the causal relationship are possible: none, remote, possible, probable and not assessable. If, in any individuals, the same adverse event occurs at several investigational times, then the adverse event in question must be documented and assessed anew each time.

Participants will be monitored by study staff for 30 min after immunization for any immediate adverse events and by the subjects themselves using a symptom diary for 28 days post immunization. Participants will be instructed to assess specific injection site adverse events (redness, swelling, warmth, tenderness, pain with arm movement) and systemic adverse events (chills, headache, muscle aches, fatigue, nausea, vomiting, diarrhoea, joint pain), all reports of other adverse events will be collected and categorized by body system. Adverse events will be collected by study personnel at each visit.

10.1.5 Definitions of adverse events

Probable - A clinical event, including laboratory test abnormality, with a reasonable time sequence to administration of the drug, unlikely to be attributed to concurrent disease or other drugs or chemicals, and which follows a clinically reasonable response on withdrawal. Rechallenge information is not required to fulfil this definition.
Possible - A clinical event, including laboratory test abnormality, with a reasonable time sequence to administration of the drug, but which could also be explained by concurrent disease or other drugs or chemicals. Information on drug withdrawal may be lacking or unclear.
Remote - A clinical event, including laboratory test abnormality, with an improbable time sequence to drug administration and in which other drugs, chemicals, or underlying disease provide a plausible explanation.
None - A clinical event for which sufficient information exists to indicate that the aetiology is unrelated to the suspected drug. This may include: (1) the existence of a clear alternative explanation (for example, mechanical bleeding at surgical site); or (2) non-plausibility (for example, the subject is struck by an automobile at least where there

is no indication that the drug caused disorientation that may have led to the event, or cancer developing a few days after drug administration).
Not assessable - A clinical event, including laboratory test abnormality, reported as an adverse reaction, about which more data is essential for a proper assessment or the additional data is under examination.

Factors to be considered include the following:

1. The temporal sequence from drug administration: The event should occur after the drug is given. The length of time from drug exposure to event should be evaluated in the clinical context of the event.
2. Recovery on discontinuation (dechallenge), recurrence on re-introduction (rechallenge): Individuals's response after drug discontinuation (dechallenge) or individual's response after drug re-introduction (rechallenge) should be considered in the view of the usual clinical course of the event in question.
3. Underlying, concomitant, intercurrent diseases: Each report should be evaluated in the context of the natural history and course of the disease being treated and any other disease the individuals may have.
4. Concomitant medication or treatment: The other drugs the individual is taking or the treatment the individual receives should be examined to determine whether they might be recognised to cause the event in question.
5. Known response pattern for this class of drug: Clinical/preclinical.
6. Exposure to physical and/or mental stresses: The exposure to stress might induce adverse changes in the recipient and provide a better, more logical explanation for the event.
7. The pharmacology and pharmacokinetics of the test drug: The pharmacokinetic properties (absorption, distribution, metabolism and excretion) of the test drugs the individual is taking, coupled with the individual's unique pharmacodynamics should be considered.

10.1.6 Intensity (severity) of the adverse event

In addition to assessing the relationship of the administration of the investigational product to adverse events, an assessment is required of the intensity (severity) of the event. The following classification should be used for events which do not have a grading in the CTC categories:
Mild: An adverse event which is usually transient in nature and generally not interfering with normal activities. The individuals may experience slight discomfort.
Moderate: An adverse event causing some limitation of usual activities that is sufficiently discomforting.
Severe: An adverse event causing inability to carry out usual activities. The individuals may experience intolerable discomfort or pain.
Life-threatening: An adverse event presenting an immediate risk of death from the event as it occurred.

10.2 Procedures for reporting SAEs

Any clinical adverse event or abnormal laboratory test value that is serious (including death, overdose or congenital anomaly) occurring during the course of the study, irrespective of the treatment received by the individuals, must be reported by the

Investigator to the Sponsor, and where relevant the Site Ethics Committee within one working day of knowledge of occurrence.

Following a report by phone, written information has to be sent by fax. For all reports, the form “Alert Report for SAEs” must be used. The adverse event itself must be documented and medically assessed by the investigator and the outcome described in the section of the CRF reserved for this purpose. Such preliminary reports will be followed by detailed descriptions later that will include copies of hospital case reports, autopsy reports and other documents when requested and applicable. This additional information will be requested, if necessary, by the responsible monitor within 5 days of receipt of the alert report. This is to ensure that the initial reporting of serious adverse reactions is made to regulatory authorities within the required time period. For a follow-up report to the authorities, the monitor may be required to collect further information for a final evaluation of the case. Reporting to the Health Authorities will be the responsibility of the Investigator.

10.3 Monitoring of individuals with adverse events

Each individual must be carefully monitored for adverse events. Assessment must be made of the seriousness, intensity and relationship to the administration of the study drug. In case of an adverse event, the investigator will decide if the study will be continued or terminated for the individuals. In case of an SAE, the study drug will be stopped, the individuals will receive appropriate treatment, and the individuals will be closely observed until reversal of the event and stabilization of the subject.

10.4 Procedures to be followed in the event of abnormal laboratory test values

In the event during the study of unexplained abnormal laboratory test values, that are considered by the investigator to be clinically significant, the tests should be repeated immediately and followed up until they have returned to the normal range and/or an adequate explanation of the abnormality is found. If a clear explanation is established it should be recorded on the CRF.

11. PRACTICAL CONSIDERATIONS AND STUDY MANAGEMENT

11.1 Study documentation, and record keeping

11.1.1 Investigator’s Files/Retention of Documents

No study document should be destroyed within 15 years of the completion of the Study. The main objective is to obtain a complete documentation of each individual in the Study.

11.1.2 Case report forms (CRF)

The data recorded in the course of this study shall be documented on the CRFs and (as necessary) on the form “Alert Report for SAEs”, that have been specially compiled for this clinical study. They shall then be recorded, evaluated, and stored in anonymous form in accordance with the data protection regulations. The CRFs must be filled in completely and legibly (with either black or blue ballpoint pen acceptable for use on official documents). Any amendments and corrections shall be undertaken and countersigned by the investigator or co-investigator, stating the date of the amendment/correction. Errors must remain legible and may not be deleted with correction aids. The investigator must state his/her reasons for the corrections

of important data. All data must correspond exactly with data recorded in the original patient files. Data to be recorded directly on the CRFs (that is, no prior written or electronic record of data, such as urinalysis dipstick results) and to be considered as source data will be identified as such. Wherever possible, all data should be recorded on the source notes. In the case of missing data/remarks, the entry spaces provided for in the CRFs should be cancelled so as to avoid unnecessary follow-up inquiries. This is particularly important when CRFs are illegible or when errors in data transcription are suspected. In the case of special problems and/or governmental queries or requests for audit inspections, it is also necessary to have access to the complete study records, provided that patient confidentiality is protected. The CRFs are documents and must be suitable for submission to authorities.

11.2 Monitoring of study

It is agreed that the course of the clinical study shall be followed up at regular intervals by the responsible Medical Advisor and by Clinical Research Associates. They, as well as the competent supervising authority, are permitted to inspect the study documents (study protocol, CRFs, study drugs, original study-relevant medical records/files). All patient data shall be treated confidentially. The study protocol, each step of the data-recording procedure, and the handling of the data as well as the study report shall be subject to Clinical Quality assurance. Audits can be conducted to assure the validity of the study data.

11.3 Amendments to the study protocol

Changes to the study protocol must take the form of written study protocol amendments. These shall require approval of all persons responsible for the study. All major protocol modifications must be submitted to the appropriate Ethics Review Committee, or its nominee, in accordance with local requirements, before the changes can be implemented. All major protocol amendments require written approval by the Ethics Committee prior to implementation. Modifications which eliminate an apparent immediate hazard to patients do not require pre-approval by the Ethics Review Committee.

11.4 Deviations from the study protocol

Deviations from the study protocol - especially the prescription of doses not scheduled in the study protocol, other modes of administration, other indications, and longer treatment periods are not permitted.

11.5 Discontinuation of study subjects

According to the Declaration of Helsinki, subjects may withdraw from the study at any time for any reason. The reason for withdrawal will be recorded in the clinical records and the CRF. Potential reasons for withdrawal are:

a. Significant adverse events, for example:
    i. Serious toxic or allergic reaction.
    ii. Intercurrent diseases.
    iii. Aggravation of a concomitant disease.
    iv. Serious laboratory abnormality.
b. Withdrawal of consent.
c. Protocol violations.

Patients who are withdrawn from the study should undergo the follow-up procedures as specified for early termination of the study.

11.6 Study discontinuation

This study may be terminated prematurely if in the best interests of patients, or justified on either medical or ethical grounds. In terminating the study, the investigator will ensure that adequate consideration is given to the protection of the patients' interests.

11.7 Confidentiality statement

The investigator must assure that patients' confidentiality will be maintained and that their identities are protected from unauthorized parties. On CRFs or other documents submitted to other bodies, patients should not be identified by their names, but by an identification code. The investigator should keep a patient enrolment log showing codes, names and addresses. Patients' written consent forms should be maintained by the investigator in strict confidence.

11.8 Study report and publication policy

In recognition of the importance of communicating basic scientific and clinical results the study results will be sought to be published in peer reviewed scientific journals and at seminars or conferences. A report will be prepared at the end of the clinical trial. The information in this report will be available for publication. Upon ethics approval the Study Protocol will be entered into a publicly accessible clinical trials database.

11.9 Anticipated individuals recruitment and duration of study

It is expected to enrol the first subject in March 2012. The final subject is due to enter the study in September 2012. Each individual will be followed up for up for a minimum of one month and up to 12 months. The minimum follow-up for the final individual is therefore expected to be in September 2013. The above timelines are based on reasonable planning expectations.

12. ETHICAL ASPECTS AND GOOD CLINICAL PRACTICE COMPLIANCE

The study will be conducted according to the International Conference on Harmonisation (ICH) guidelines for GCP and the Declaration of Helsinki (Edinburgh, Scotland, October 2000) and Australian NH&MRC Guidelines.

12.1 Ethical conduct of the study

All clinical work conducted under this protocol is subject to Good Clinical Practice (GCP) rules, and this may include inspection by the study monitor and/or Ethical Review Committee at any time. The investigator must adhere to the above principles in addition to any applicable local regulations. After completion of the study, the investigator must inform the appropriate Ethics Committee (EC) that the trial is concluded. Every serious or unexpected adverse event that might affect the patients' safety must be brought to the EC's attention by the investigator.

12.2 Protocol adherence

The protocol must be read thoroughly and the instructions must be followed. Any deviations should be agreed to by both the sponsor and the investigator, with appropriate written protocol amendments made to reflect the changes agreed upon.

Where the deviation occurs for the well-being of the individuals, the monitor must be informed and a course of action agreed upon.

12.3 Informed consent and individual information

Informed consent must be obtained from the individuals by the investigator or their delegate in accordance with the Declaration of Helsinki and national laws and regulations.

14. GLOSSARY

ALAT	Alanine aminotransferase
ASAT	Aspartate aminotransferase
ATC	Anatomic Therapeutic Class
HCG	Beta-human chorionic gonadotrophin
CAF	Cyclophosphamide, Adriamycin and 5-Fluorouracil
CMF	Cyclophosphamide, Methotrexate and 5-Fluorouracil
CMI	Cell Mediated Immunity
CNS	Central nervous system
COPD	Chronic Obstructive Pulmonary Disease
CRA	Clinical Research Assistant
CR	Complete response
CHD	Chronic Heart Disease
CRF	Chronic Renal Failure
CLINICAL RF	Case report form
CTC	Common toxicity criteria
CTL	Cytotoxic T Lymphocytes
DLTs	Dose limiting toxicities
DMSO	Dimethylsulfoxide
VACCINE	Deoxyribonucleic Acid
EC	Ethics Committee
ECG	Electrocardiogram
EDTA	Ethylenediaminetetraacetic acid
ELISA	Enzyme-linked immunosorbent assay
ELISPOT	Enzyme Linked Immuno Spot
EMEA	European Agency for the Evaluation of Medical Products
EU	European Union
FCS	Foetal Calf Serum
FDA	Food and Drug Administration
GCP	Good Clinical Practice
GGT	gamma-Glutamyl transpepsidase
HI	Haemagglutinin inhibition
i.m.	Intra muscular
ICH	International Conference on Harmonisation
IFN gamma	Interferon Gamma
Ig	Immunoglobulin
IRB	Institutional Review Board
ITT	Intention-to-treat
LD	Longest Diameter

MedDRA	Medical Dictionary for Regulatory Activities Terminology
MHC	Major Histocompatibility Complex
MTD	Maximum tolerated dose
MUGA	Multi-Gated Acquisition
PBMC	Peripheral Blood Mononuclear Cells
PBS	Phosphate-buffered saline
PD	Progressive disease
PHA	Phytohemagglutinin Antigen
PP	Per protocol
PPD	Purified Protein Derivative of M. Tuberculosis
PR	Partial Response
SAE	Serious adverse event
SD	Stable disease
TRIS	Tris(hydroxymethyl)aminomethane
UNL	Upper normal limit
WBC	White blood count
WHO	World Health Organisation
WHO-DD	WHO drug dictionary

15. REFERENCES:

1. Lowering the age for routine influenza vaccination to 50 years: AAFP leads the nation in influenza vaccination policy. Zimmerman, R.K. (1999) American Family Physician, 60, 2061-6, 2069-70.

2. Clinical effectiveness of influenza vaccination in persons younger than 65 years with high-risk medical conditions. Hak, E. et al. (2005) Archives of Internal Medicine, 165, 274-280.

3. Vaccines for preventing influenza in people with asthma. Cochrane Database of Systematic Reviews. CJ Cates, TO Jefferson, AI Bara, BH Rowe.

4. Vaccines for preventing influenza in people with cystic fibrosis. Cochrane Database of Systematic Reviews. P Bhalla, A Tan, R Smyth.

5. Influenza vaccine for patients with chronic obstructive pulmonary disease. Cochrane Database of Systematic Reviews, CD002733. Poole, P. J. et al (2000).

6. Influenza and atherosclerosis: vaccination for cardiovascular disease prevention. Madjid, M. et al. (2005) Expert Opin Biol Ther, 5, 91-96.

7. Rivetti D, Jefferson T, Thomas R, Rudin M, Rivetti A, Di Pietrantonj C, Demicheli V. Vaccines for preventing influenza in the elderly. Cochrane Database of Systematic Reviews 2006, Issue 3. Art. No.: CD004876. DOI: 10.1002/14651858.CD004876.pub2

Amendments to the study protocol

Changes to the study protocol must take the form of written study protocol amendments. These shall require approval of all persons responsible for the study. All protocol modifications must be submitted to the appropriate Ethics Review Committee, or its nominee, in accordance with local requirements, before the changes can be implemented. All protocol amendments require written approval by the Ethics Committee prior to implementation. Modifications which eliminate an apparent immediate hazard to patients do not require pre-approval by the Ethics Review Committee.

This study is to be conducted in compliance with the NH&MRC National Statement on ethical conduct in research involving humans
